# A bioinspired microfluidic wearable sensor for multiday sweat sampling, transport, and metabolic analysis

**DOI:** 10.1126/sciadv.adw9024

**Published:** 2025-08-13

**Authors:** Soyoung Shin, Ruixiao Liu, Yiran Yang, José A. Lasalde-Ramírez, Gwangmook Kim, Chihyeong Won, Jihong Min, Canran Wang, Kexin Fan, Hong Han, Chibuike Uwakwe, Wenzheng Heng, Tzung K. Hsiai, Zhaoping Li, John D. FitzGerald, Wei Gao

**Affiliations:** ^1^ Andrew and Peggy Cherng Department of Medical Engineering, Division of Engineering and Applied Science, California Institute of Technology, Pasadena, CA 91125, USA.; ^2^ Department of Medicine, UCLA David Geffen School of Medicine, Los Angeles, CA 90095, USA.

## Abstract

Wearable sweat sensors enable noninvasive real-time biochemical monitoring, holding immense potential for personalized health care applications. However, achieving prolonged and reliable sweat sampling, along with stable biochemical analysis, remains challenging due to inconsistent secretion, rapid evaporation, and the reliance on external stimulation. Here, we present BMS^3^, a bioinspired microfluidic wearable sweat sensor system designed for multiday continuous metabolic monitoring. BMS^3^ integrates hierarchically graded microchannels and superhydrophobic-superhydrophilic Janus membranes, inspired by pitcher plant trichomes and lotus leaves to enable efficient low volume sweat collection, transport, and renewal. A miniaturized carbachol gel–based iontophoresis module autonomously induces localized sweat secretion. Furthermore, the microfluidic design sustains sweat sampling for over 2 days from a single iontophoresis session, eliminating the need for physical exertion. In vitro and in vivo studies in healthy participants and patients with gout demonstrate BMS^3^’s capability for continuous metabolic monitoring. By simultaneously tracking uric acid, xanthine, and alcohol levels, it effectively differentiates normal and pathological states while delivering timely therapeutic feedback.

## Teaser

A bioinspired wearable sensor enables multiday sweat analysis for real-time metabolic health and personalized disease monitoring.

## INTRODUCTION

Wearable sensors have the potential to set the foundation for precision medicine by enabling noninvasive and continuous monitoring of the body’s biochemical landscape. Among various biofluids, sweat offers a rich source of biochemical information that can be accessed conveniently and noninvasively, making it particularly attractive for real-time health monitoring (*1*–*8*). Unlike blood, interstitial fluid, tears, saliva, and urine—that require invasive or cumbersome sampling techniques—sweat can be easily collected using a sensor patch placed on the skin. By continuously tracking molecular biomarkers in sweat, wearable sensors provide valuable insights into physiological and pathological conditions, facilitating precision medicine through personalized monitoring of fitness, health conditions, and disease progression.

Over the past decade, substantial advancements in wearable sweat sensors have enabled the detection of diverse biomarkers, including electrolytes, metabolites, nutrients, drugs, hormones, and proteins (*2*). These sensors have been explored for a wide range of biomedical applications, such as fitness tracking (*9*–*11*), nutritional assessment (*12*–*16*), disease management (*17*–*19*), stress evaluation (*20*–*24*), fertility monitoring (*25*), and therapeutic drug tracking (*26*–*28*). Despite their potential, key challenges persist in achieving reliable, long-term sweat sampling and analysis—which are critical for personalized health care applications (*2*, *29*).

Traditional sweat collection methods rely on physical exertion or external heat stimulation, which can be impractical for continuous and controlled data acquisition. As a result, there is increasing interest in monitoring naturally secreted sweat at rest, particularly in high–sweat gland density regions like the fingertips, using point-of-care analysis (*30*–*32*). However, the effectiveness of this approach is limited by inconsistent, slow, and unstable nature of sweat secretion in real-world applications, which is influenced by environmental factors, physical activity levels, and individual physiological variability.

Iontophoresis (IP)—a technique that delivers a cholinergic drug into the skin via a small electrical current—offers a promising alternative for localized sweat induction without the need for exercise or temperature modulation (*24*, *33*–*35*). Among cholinergic agents, carbachol stands out due to its ability to activate both muscarinic and nicotinic receptors, triggering the sudomotor axon reflex and promoting sweat secretion over a larger skin surface area (*12*, *35*, *36*). Moreover, sweat induced by carbachol maintains a relatively stable pH with minimal fluctuations and therefore serves as an ideal medium for molecular analysis. Although carbachol IP sustains sweat secretion over multiple days due to carbachol’s resistance to enzymatic degradation and retention in the skin, the induced sweat flow rate declines substantially within a few hours in practical wearable applications. Existing sweat sensors typically provide continuous monitoring for only a limited duration per IP session (*21*, *36*), limiting sampling efficiency and the sensor’s ability to deliver timely health insights. Increasing drug dosage or IP frequency could theoretically extend sweat production; however, concerns over drug safety, potential skin irritation, and hydrogel stability present barriers to daily use.

**Fig. 1.**
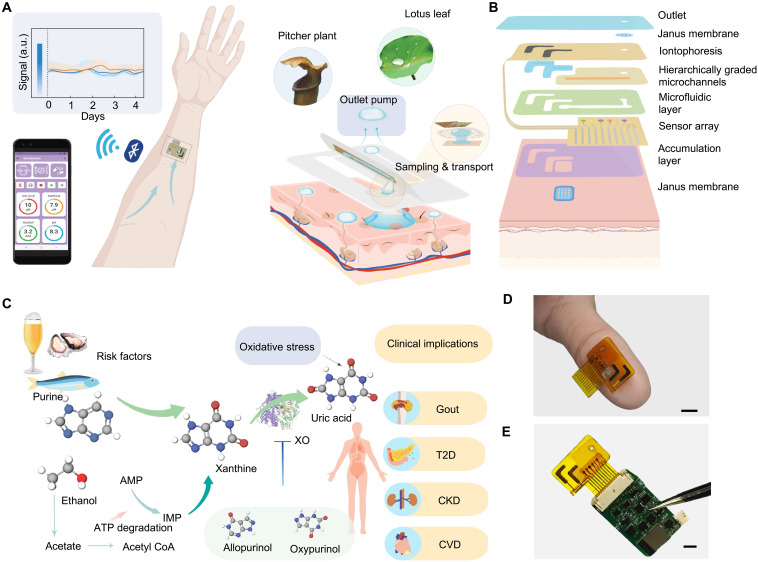
BMS^3^—A bioinspired microfluidic sweat sensor system. (**A**) Overview of BMS^3^, featuring microfluidic structures inspired by the lotus leaf and pitcher plant’s trichomes for extended sweat sampling, transport, and multiday sweat analysis. a.u., arbitrary units. (**B**) Schematic of the layered structure of the BMS^3^, including the IP module, superhydrophobic-superhydrophilic Janus membranes, hierarchically graded microchannels, microfluidic transport layers, and electrochemical sensor array. (**C**) Mechanistic illustration of the purine metabolism and its clinical implications: High-purine diets and alcohol consumption elevate circulating purine metabolites (xanthine and uric acid), contributing to oxidative stress and increasing the risk of metabolic disorders such as gout, type 2 diabetes (T2D), chronic kidney disease (CKD), and cardiovascular disease (CVD). Xanthine oxidase (XO) catalyzes the conversion of xanthine to uric acid, whereas allopurinol and oxypurinol inhibit this enzymatic activity. Furthermore, the metabolism of alcohol triggers adenosine triphosphate (ATP) degradation pathways, converting ATP to adenosine monophosphate (AMP). A portion of the AMP is then further metabolized into inosine monophosphate (IMP), which breaks down into hypoxanthine and, ultimately, uric acid. CoA, coenzyme A. (**D** and **E**) Photographs of BMS^3^ prototypes, including a disposable sensor patch applied on the thumb (D) and a fully integrated wireless BMS^3^ device (E). Scale bars, 5 mm.

To address these challenges, we introduce BMS^3^ (bioinspired microfluidic sweat sensor system), a wearable platform designed for extended sweat sampling, transport, and multiplexed metabolic analysis over multiple days (Fig. 1A). This system integrates a pair of superhydrophobic-superhydrophilic silica nanoparticle–coated Janus membranes and hierarchically graded microchannels, structurally inspired by the lotus leaf and pitcher plant trichomes, respectively, to enable continuous, efficient collection, transport, and refreshing of small-volume sweat samples (Fig. 1B). By incorporating a minimized carbachol gel–based IP module, BMS^3^ autonomously induces localized sweat secretion at rest with a single short-term IP session. The Janus membrane harvests and guides the sweat from the skin into the microfluidic layer, where hierarchical microchannels facilitate rapid transport to the sensing array for analysis, even under low sweat flow conditions. An additional Janus membrane at the outlet functions as a passive pump, ensuring continuous sweat renewal for uninterrupted monitoring. This bioinspired design enables real-time molecular analysis over extended periods (up to several days) with a single IP session, providing a robust and reliable platform for continuous molecular monitoring.

To validate its practical biomedical utility, we developed a multimodal wearable device capable of multiplexed analysis of key lifestyle-associated metabolic biomarkers, including purine metabolites (uric acid and xanthine) and alcohol—critical indicators of metabolic pathways and overall metabolic health, particularly for gout management (Fig. 1C). The miniaturized flexible microfluidic sensor patch, fabricated via inkjet printing coupled with laser engraving (fig. S1), seamlessly interfaces with a custom-designed printed circuit board (PCB) for wireless signal processing and communication, enabling real-time on-body monitoring (Fig. 1, D and E). Through a series of in vitro tests and in vivo studies conducted in healthy participants and patients with gout, we demonstrate that BMS^3^ realizes continuous, noninvasive monitoring of metabolic fluctuations during daily activities. The system effectively differentiates normal from pathological metabolic states while providing timely feedback on therapeutic efficacy, underscoring its potential for personalized disease management and intervention.

## RESULTS

### Design and characterization of the multiplexed sensor patch

**Fig. 2.**
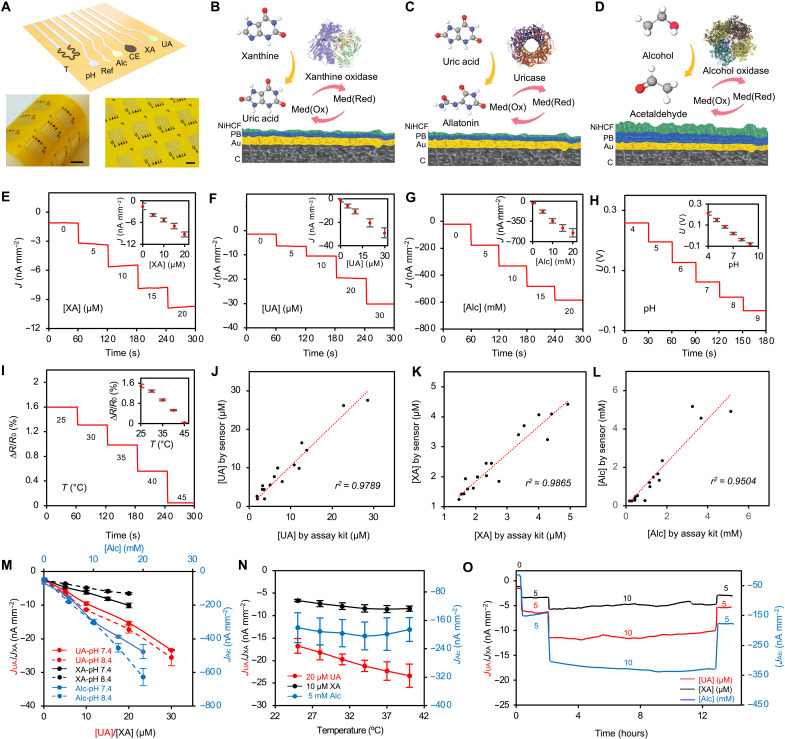
Design and characterization of the multiplexed sensor patch. (**A**) Schematic and images of the inkjet-printed flexible sensor array. XA, xanthine; UA, uric acid; Alc, alcohol; CE, counter electrode; Ref, reference electrode; T, temperature. Scale bars, 5 mm. (**B** to **D**) Electrode design and electrochemical sensing mechanisms for enzymatic detection of xanthine (B), uric acid (C), and alcohol (D). Med(Ox) and Med(Red) represent the oxidized and reduced states of the mediator, respectively. C, carbon; PB, Prussian blue; NiHCF, nickel hexacyanoferrate. (**E** to **G**) Amperometric responses of the xanthine (E), uric acid (F), and alcohol (G) sensors in PBS (1x, pH 9.4) with physiologically relevant analyte concentrations. Insets: Calibration plots with a linear fit. *J*, current density. Error bars represent the SD of the mean from nine sensors. (**H** and **I**) Dynamic responses of pH (H) and temperature (I) sensors under physiologically relevant conditions. Error bars represent the SD of the mean from six sensors. (**J** to **L**) Sensor validation against commercial assay kits for analyzing uric acid (J), xanthine (K), and alcohol (L) in human sweat samples. Data are based on 16 sweat samples collected from human subjects. (**M** and **N**) Calibration plots of xanthine, uric acid, and alcohol sensors under varying pH (M) and temperature (N) conditions. Error bars represent the SD of the mean from five sensors. (**O**) Operational long-term stability of the enzymatic biosensors.

Figure 2A illustrates an example sensor array of BMS^3^ designed for gout management. This array features three working electrodes for the selective analysis of key gout-related biomarkers—xanthine, uric acid, and alcohol—to enable comprehensive profiling of purine metabolism. In addition, we also integrated pH and temperature sensors to facilitate real-time calibration, ensuring accurate and stable biochemical readings. We selected these biomarkers based on their crucial role in gout pathophysiology. Specifically, xanthine serves as a precursor in purine metabolism, and its accumulation indicates inhibition of xanthine oxidase activity (*37*). Uric acid, the final oxidation product of purines, is the primary marker of hyperuricemia, a direct cause of gout (*38*). Blood uric acid levels are determined by a combination of factors, including age, diet, medications, and genetics. Alcohol increases serum urate levels by accelerating purine degradation and reducing renal excretion, making it a key lifestyle factor in gout management (*39*, *40*). By tracking these biomarkers simultaneously, the sensor array provides a comprehensive and personalized therapeutic guidance for patients with gout. The multiplexed and mechanically flexible sensor patch can be mass-produced with inkjet printing on a polyimide (PI) substrate. Surface morphology characterization using cross-sectional scanning electron microscopy (SEM) (fig. S2) and electrochemical analysis using cyclic voltammetry (CV) (fig. S3) demonstrate that the printed gold nanoparticle–based working electrodes exhibit a substantially higher electrocatalytic surface area compared to electroplated or evaporated gold electrodes.

To achieve selective analysis of xanthine, uric acid, and alcohol, we functionalized enzymatic electrochemical electrodes with xanthine oxidase, uricase, and alcohol oxidase, respectively (Fig. 2, B to D). These enzymes efficiently catalyze reactions of their target molecular and oxygen, generating hydrogen peroxide (H_2_O_2_) as a by-product. We used Prussian blue (PB) as a redox mediator to facilitate H_2_O_2_ reduction at near-zero potential, minimizing interference from other electroactive species. However, PB’s poor operational stability, caused by the solubilization of ferric hexacyanoferrate under alkaline conditions, limits long-term use (*21*). To enhance stability and enable continuous monitoring, we introduced a nickel hexacyanoferrate (NiHCF) stabilizing layer on top of PB (*21*). We optimized the deposition method for each analyte based on expected physiological concentration ranges: a thin PB layer applied via pulsed deposition enhanced sensitivity for xanthine and uric acid (micromolar range), whereas a thick PB layer applied via CV improved performance for alcohol detection (millimolar range) (fig. S4).

We evaluated the electrochemical performance of the xanthine, uric acid, and alcohol sensors using chronoamperometry in phosphate-buffered saline (PBS) (Fig. 2, E to G). For this in vitro test, we selected a pH of 9 based on the average pH of carbachol-induced sweat (fig. S5). The sensors demonstrated high stability, selectivity, and reproducibility, with a linear correlation between current output and target analyte concentrations across physiologically relevant ranges (Fig. 2, E to G, and figs. S6 and S7). The sensitivities for xanthine, uric acid, and alcohol sensors were 0.37 nA μM^−1^ mm^−2^, 0.93 nA μM^−1^ mm^−2^, and 26.2 nA mM^−1^ mm^−2^, respectively. In addition, they exhibited long-term storage stability for over 4 weeks at 4°C (fig. S8).

Given that sweat pH and skin temperature vary between individuals and substantially affect enzymatic sensor responses, the sensor patch integrates a polyaniline (PANI)–based potentiometric pH sensor and an inkjet-printed resistive temperature sensor. The pH sensor exhibited a linear response to solution pH, with a sensitivity of 59.5 mV pH^−1^, whereas the temperature sensor demonstrated a linear resistance-temperature correlation across the physiologically relevant range of 25° to 45°C, with a relative sensitivity of 0.138% °C^−1^ (Fig. 2, H and I, and fig. S9).

We validated the accuracy of enzymatic xanthine, uric acid, and alcohol sensors for human sweat analysis by comparing their readings against commercially available assay kits. Results showed strong agreement between the two approaches (Fig. 2, J to L). To enhance analytical accuracy during in vitro and on-body tests, we characterized the influence of pH and temperature on sensor performance electrochemically (Fig. 2, M and N) and used obtained information for real-time calibration of enzymatic sensor readings.

Last, we assessed the long-term stability of the sensors over 14 hours of continuous measurement under varying analyte concentrations (Fig. 2O). The results demonstrated that all sensors maintained stable responses throughout testing, with rapid and selective responses to target analyte changes, while remaining unaffected by non–target analyte fluctuations.

### Characterization of bioinspired microfluidics for efficient fluid harvesting and transport

**Fig. 3.**
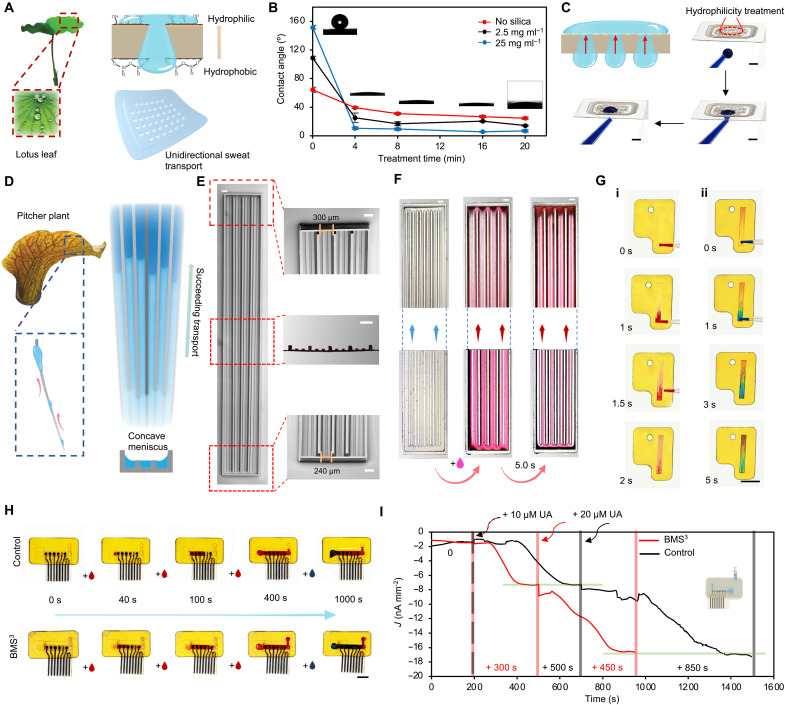
Design and characterization of the bioinspired microfluidics for low-volume sweat harvesting and transport. (**A**) Schematic illustration of the superhydrophobic-superhydrophilic silica nanoparticle–coated Janus membrane, inspired by the lotus leaf, enabling unidirectional liquid transport. (**B**) Effect of O_2_ plasma etching time on the contact angle of membrane surfaces coated with varying silica concentrations. (**C**) Unidirectional flow transport via the Janus membrane. Scale bars, 2 mm. (**D**) Schematic of the hierarchically graded microchannels, inspired by the pitcher plant, for efficient sweat transport. (**E**) Images of the hierarchically graded microchannels, including SEM images of the full structure, top and bottom features, and an optical cross-sectional image. Scale bars, 200 μm. (**F**) Images of the flow test, demonstrating rapid unidirectional transport within the microchannel using 0.2 μl of pink-dyed water. Scale bars, 200 μm. (**G**) Demonstration of unidirectional liquid transport in the microchannel, where 0.2 μl of red dye is followed by 0.2 μl of blue dye. Scale bar, 5 mm. (**H**) Comparison of the flow transport between control microfluidics (without bioinspired structures) and the BMS^3^ patch, which incorporates Janus membranes at the inlet and outlet alongside hierarchically graded microchannels. Each drop represents 0.5 μl of dye. Scale bar, 5 mm. (**I**) Amperometric responses of uric acid sensors to dynamic uric acid level changes, comparing the control microfluidics and the assembled BMS^3^ patch under a flow rate of 0.1 μl min^−1^.

The microfluidic system of BMS^3^ integrates two Janus membranes at the inlet and outlet alongside hierarchically graded microchannels to realize efficient low-volume sweat harvesting, transport, and renewal for real-time dynamic wearable biosensing. Inspired by the lotus leaf (*41*–*45*), the Janus membrane enables enhanced sweat harvesting from the skin and unidirectional low-volume sweat transport in wearable biosensors (Fig. 3A). This dual-functional membrane leverages its dual-sided properties of superhydrophilicity and superhydrophobicity for effective sweat absorption and retention. The superhydrophilic side, created via O_2_ plasma–treated silica nanoparticles (fig. S10 and movie S1), rapidly absorbs sweat from the skin and directs it into the sensor system, mimicking the water-attracting properties of the lotus leaf’s inner surface. Meanwhile, the superhydrophobic side, coated with untreated silica nanoparticles, prevents premature sweat evaporation and loss, ensuring a stable environment for biomarker detection, resembling the water-repellent outer surface of the lotus leaf.

To optimize performance, we evaluated various silica concentrations and O_2_ plasma treatment durations. A concentration of 25 mg ml^−1^ was selected to achieve superhydrophobicity (contact angle > 150°), whereas a 16-min plasma treatment rendered the opposing surface superhydrophilic (Fig. 3B, fig. S11, and movie S1). Wettability and shelf stability assessments confirmed the Janus membrane’s long-term durability over 5 days (fig. S12). Unidirectional transport of sweat through the Janus membrane was demonstrated by applying blue ink at the hydrophobic bottom, which seamlessly transitioned to the hydrophilic top, forming a stable meniscus (Fig. 3C and fig. S13). This mechanism enables the Janus membranes to function as both a sweat harvester at the inlet and an outlet pump, facilitating continuous sweat transport and refreshment. The system’s design parameters have been carefully optimized to ensure stable meniscus formation, which maintains consistent contact with the microchannel to facilitate rapid fluid transport (fig. S14). Notably, the optimized Janus membrane forms a stable meniscus that remains pinned under continuous droplet addition, mimicking the uninterrupted flow of sweat without depinning or backflow (fig. S15).

Although Janus membrane enables sweat harvesting, efficient sweat transport through the sensors is achieved via a hierarchically graded microchannel system (*46*, *47*), inspired by the pitcher plant’s trichomes (Fig. 3D and fig. S16). Two key design features were incorporated (Fig. 3E): (i) dual-height ribs for enhanced liquid film formation, mimicking the trichome structure, and (ii) a geometric gradient resembling a cactus spine, with narrow-to-wide channel widening, directing sweat flow efficiently toward the sensor. To optimize this architecture, we systematically evaluated five microchannel configurations with varying rib heights and width gradients (fig. S17). Impedance-based flow characterization showed that a design with moderate rib height and lateral asymmetry achieved the most consistent and rapid wetting behavior, leveraging Laplace pressure differentials to sustain unidirectional flow and minimize backflow (fig. S18, A to C).

To further promote directional transport, we integrated a Janus membrane with matching asymmetric wettability at the outlet. Impedance monitoring (fig. S18D) confirmed that this configuration maintained steady flow and prolonged wetting, highlighting the outlet’s role as a passive fluidic diode that reinforces unidirectional fluid movement.

This bioinspired structure ensures fast and unidirectional low volume sweat transport, optimized for continuous monitoring (Fig. 3F and movie S2). Considering that continuous dynamic sweat monitoring requires efficient sweat refreshment, we evaluated transport efficiency by introducing red ink (0.2 μl) in an antigravity direction. The ink was fully transported within 2 s, demonstrating rapid and effective fluid movement. A subsequent application of blue ink confirmed seamless transition (Fig. 3G). In addition, long-term durability and unidirectional wettability were validated through 5-day testing (fig. S19).

Comparative tests confirmed that control systems lacking bioinspired structures failed to transport sweat efficiently, whereas BMS^3^ immediately formed a thin liquid film, allowing rapid transport (Fig. 3H). Even when fully occupied, BMS^3^ continued to facilitate efficient dye movement, whereas the control exhibited stagnation. To assess the system’s biomarker transport efficiency, we further conducted a low-flow biomarker comparison using uric acid sensors (Fig. 3I). Both systems were first stabilized in PBS and then switched to a 10 μM uric acid solution. BMS^3^ reached the target current density in ~300 s, whereas the control required an additional 200 s. At 20 μM uric acid, this performance gap widened further, consistent with observations from Fig. 3H, where the control system struggled to transport fresh analytes after initial channel filling, unlike BMS^3^, which maintained smooth biomarker transition.

These results demonstrate that our bioinspired Janus membrane and hierarchical microchannel system substantially enhance sweat harvesting, transport, and biomarker detection, ensuring continuous, real-time metabolic monitoring in wearable biosensors.

As the BMS^3^ patch is designed for long-term on-skin wearable use, evaluation of its cytocompatibility and biocompatibility is critical. We tested cytocompatibility by culturing cells in media containing extracts from the device, followed by viability assessment using standard live/dead staining and 3-[4,5-dimethylthiazol-2-yl]-2,5 diphenyl tetrazolium bromide (MTT) assays (fig. S20). We observed consistently high cell viability over a 7-day period through both fluorescence imaging and absorbance measurements, confirming the patch’s excellent cytocompatibility.

### On-body evaluation of the BMS^3^ system

**Fig. 4.**
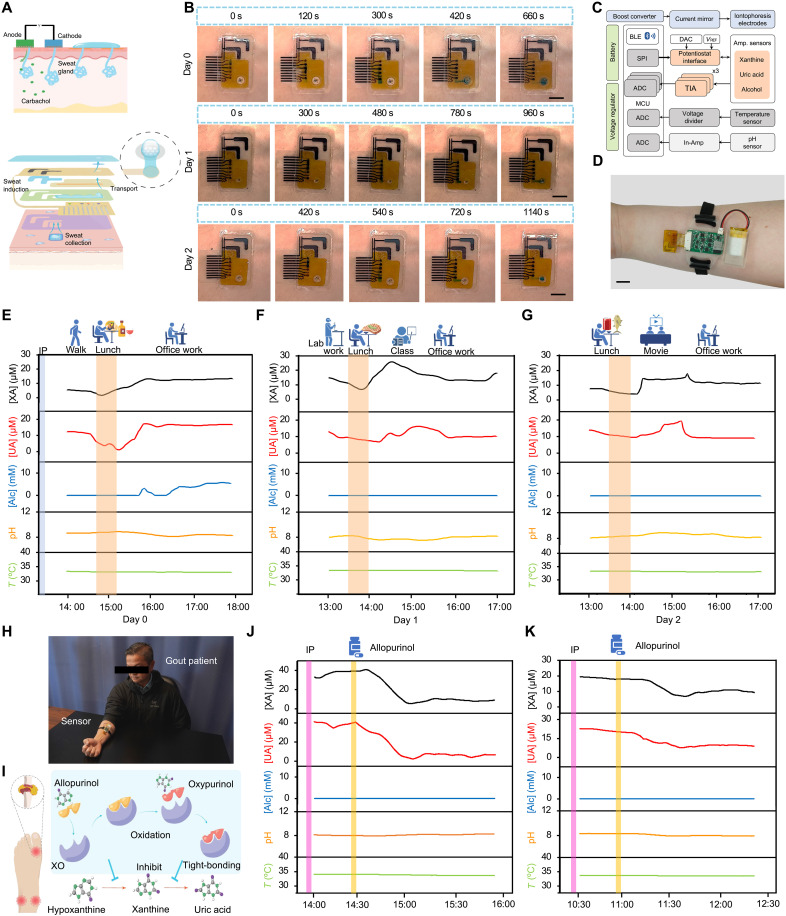
On-body evaluation of BMS^3^ in healthy participants and patients with gout. (**A**) Schematic illustration of the localized sweat induction with IP followed by microfluidic sweat harvesting and sampling. (**B**) Time-lapse visualization of sweat sampling at 8, 24, and 48 hours after IP application, demonstrating multiday sustained sweat collection. Scale bars, 5 mm. (**C**) Block diagram of the wireless electronic system of BMS^3^. ADC, analog-to-digital converter; AFE, analog front end; CA, control amplifier; DAC, digital-to-analog converter; EXA, excitation amplifier; MUX, multiplexer; TIA, transimpedance amplifier. (**D**) Photograph of a healthy participant wearing the BMS^3^ sensor patch. Scale bar, 1 cm. (**E** to **G**) Three-day multimodal biochemical monitoring in a healthy participant, tracking metabolic fluctuations during daily activities following a single IP session. (**H**) Photograph of a patient with gout undergoing sensor-based biochemical monitoring during the study after allopurinol intake for gout management. (**I**) Mechanism of allopurinol, illustrating its role as an inhibitor for XO, reducing uric acid production by blocking the conversion of hypoxanthine and xanthine. (**J** and **K**) Real-time biochemical response monitoring in two patients with gout following allopurinol intake, revealing dynamic metabolic changes in uric acid and related biomarkers while providing therapeutic feedback.

With an optimized sensor array and bioinspired microfluidics for sweat harvesting, transport, and renewal, we evaluated the multiplexed sensor patches on human participants (Fig. 4A). A small IP unit was integrated to induce localized sweat secretion, applying 100 μA for 6 min, which resulted in sweat induction within 10 min post-IP session (fig. S21). Notably, our system remains effective in sampling sweat after 2 days following a single IP session (movie S3).

To visualize sweat sampling in the BMS^3^, we introduced blue ink, using a freshly prepared passive microfluidic patch daily. Day 0 data were recorded 8 hours post-IP, whereas days 1 and 2 data were collected at 24 and 48 hours, respectively. Time-lapse imaging confirmed that the system efficiently harvested and transported sweat through the microfluidic channels to the outlet, ensuring continuous sweat refreshment (Fig. 4B). Additional tests across multiple subjects 24 hours post-IP demonstrated that the system could effectively capture and transport low sweat volumes, enabling continuous sensing under low sweat rate conditions (fig. S22). To further demonstrate versatility, the device was applied immediately before exercise, and blue dye was introduced into the microfluidic channel to visualize dynamic fluid movement. The system successfully captured exercise-induced sweat and maintained continuous flow throughout active perspiration (fig. S23).

For practical wearable monitoring scenarios, we integrated a PCB into BMS^3^ for signal processing and wireless communication (Fig. 4C and fig. S24). The compact, user-friendly design allowed placement on the inner forearm, ensuring comfort and functionality for long-term use (Fig. 4D). Before on-body testing, we established calibration curves to evaluate the sensor array’s performance with the PCB (fig. S25).

To assess the long-term usability of BMS^3^ for personalized metabolic tracking, we conducted a 3-day evaluation following a single IP session on a healthy participant, continuously monitoring biochemical fluctuations across various daily activities (Fig. 4, E to G). On day 0, the sensor data were recorded 1 hour post-IP during activities including walking, lunch (burrito + alcohol), and office work. On day 1 (24 hours post-IP), activities included lab work, lunch (red meat), classes, and office tasks. On day 2 (48 hours post-IP), participants engaged in lunch (fish + soda), movie watching, and office work. Throughout the monitoring period, the pH (~8.5) and temperature (~32°C) remained stable, whereas xanthine, uric acid, and alcohol levels exhibited distinct fluctuations linked to dietary intake. As expected, consumption of purine-rich foods such as red meats and seafood, along with high-fructose foods, led to increased uric acid production. In addition, alcohol consumption from beer and spirits exacerbated hyperuricemia risk, aligning with established metabolic pathways. To validate these findings, we monitored two additional individuals (fig. S26), confirming the effectiveness of BMS^3^ for personalized metabolic tracking in real-world scenarios. In addition, we used BMS^3^ to monitor exercise-induced sweat and evaluate metabolic responses before and after consumption of high-purine foods. This experiment further demonstrated the device’s ability to capture dynamic fluctuations in sweat metabolites under different modes of sweat induction (fig. S27).

### Clinical validation in patients with gout

To expand the clinical applicability of our biosensor, we tested two patients with gout with elevated uric acid levels, both before and after allopurinol intake (Fig. 4H). Allopurinol, a purine analog, is rapidly metabolized (by xanthine oxidase) to oxypurinol. Both the parent and metabolite inhibit xanthine oxidase, although oxypurinol provides the primary inhibition of xanthine oxidase and therefore blocks the conversion of hypoxanthine to xanthine and xanthine to uric acid (Fig. 4I) (*48*, *49*). Both patients with gout exhibited substantially elevated uric acid and xanthine levels compared to healthy individuals, reinforcing the need for uric acid regulation. Following allopurinol oral administration, both uric acid and xanthine levels dropped substantially, confirming the drug’s therapeutic effect (Fig. 4, J and K). It is important to note that, because allopurinol does not affect hypoxanthine levels, the xanthine sensor (which responds to both xanthine and hypoxanthine) remained responsive. Given the half-life of allopurinol (1 to 2 hours) and oxypurinol (18 to 30 hours) (*50*), the presence of these compounds in sweat could modulate the xanthine oxidase enzyme reaction, leading to lower xanthine sensor readings. To investigate further, we conducted a follow-up test 2 hours post-allopurinol intake in the same patient. Results showed a higher xanthine reading whereas uric acid levels remained low, suggesting that oxypurinol strongly binds to xanthine oxidase in vivo, reducing its presence in sweat and causing a slight increase in xanthine levels (fig. S28). Positive correlations between sweat and blood uric acid levels were observed in both treated and untreated patients (fig. S29). Notably, patients not receiving allopurinol exhibited substantial fluctuations in uric acid levels, whereas more stable trends were seen in those under active treatment. These findings further substantiate that BMS^3^ enables real-time, continuous metabolic monitoring, offering valuable insights into personalized disease management and therapeutic efficacy in patients with gout.

## DISCUSSION

This study presents BMS^3^, a bioinspired microfluidic wearable sweat sensor system designed for continuous, multiday metabolic monitoring. By integrating hierarchically graded microchannels and Janus membranes, inspired by pitcher plant trichomes and the lotus leaf, BMS^3^ ensures efficient, continuous, and low volume sweat sampling, transport, and renewal, overcoming the key limitations of conventional sweat sensors. The incorporation of a carbachol gel–based IP module autonomously induces localized sweat secretion at levels sufficient for biochemical analysis with a brief stimulation session. Notably, BMS^3^ supports sustained sweat collection for over 48 hours without requiring repeated stimulation—an advance not demonstrated in previous studies. The system uses a miniaturized carbachol gel patch with low dosage to safely maintain sweat flow. The inlet Janus membrane efficiently harvests sweat from the skin and forms microdroplets, which are directed into the sensing region via the hierarchical microchannel. The outlet Janus membrane then removes the analyzed sweat, preventing accumulation and allowing fresh sweat to enter, thus maintaining clean, continuous sampling.

Our results demonstrate that BMS^3^ enables the real-time, noninvasive tracking of key purine metabolites, including uric acid, xanthine, and alcohol, in sweat, providing a reliable biochemical profile for metabolic health monitoring. The wireless, fully integrated system effectively captures dynamic changes associated with dietary intake, alcohol consumption, and therapeutic drug response, ensuring its practical applicability in everyday settings. By validating the system’s performance through in vitro and in vivo studies in both healthy participants and patients with gout, we highlight its potential for personalized disease management. Clinical evaluation in patients with gout before and after allopurinol administration further underscores the clinical relevance of BMS^3^. The system successfully distinguished between normal and pathological metabolic states and provided timely therapeutic feedback by detecting changes in uric acid and xanthine levels following allopurinol intake.

Moving forward, several enhancements could further expand the impact of BMS^3^. The modular sensor platform can be adapted for additional metabolic and inflammatory biomarkers to support broader clinical applications. Incorporating artificial intelligence–driven analytics could enhance the interpretation of biochemical fluctuations, providing predictive insights for early disease intervention. Long-term longitudinal studies across diverse patient populations will be critical for validating BMS^3^ in personalized health management.

## MATERIALS AND METHODS

### Materials

Chitosan, carbachol, polyurethane (PU), agarose, glutaraldehyde (GA), potassium chloride (KCl), nickel chloride, potassium(III) ferricyanide (K_3_[Fe(CN)_6_]), bovine serum albumin (BSA), 10× PBS, polyvinyl butyral resin (BUTVAR B-98; PVB), silica nanoparticles, aniline, iron(III) chloride, sodium chloride (NaCl), sodium hydroxide (NaOH) lactic acid, alcohol oxidase (AOx) from *Pichia pastoris* (10 to 40 U mg^−1^), ethanol assay kit, and urea were obtained from Sigma-Aldrich. Hydrochloric acid (HCl), acetic acid, toluene, methanol, anhydrous ethanol, hydrogen peroxide (30%, w/v), sulfuric acid (H_2_SO_4_), *N*,*N*′-dimethylformamide (DMF), tetrahydrofuran (THF), Amplex Red Uric Acid/Uricase Assay Kit, 1*H*,1*H*,2*H*,2*H*-perfluorooctyltrichlorosilane (PFOTS; 97%), and dextrose (d-glucose) were sourced from Thermo Fisher Scientific. Carbon ink (5 wt %) was purchased from NovaCentrix, and gold ink (10 wt %) was purchased from C-INK Co. Ltd. The Xanthine/Hypoxanthine Assay Kit was purchased from Abcam. Uricase (5.6 U mg^−1^) and xanthine oxidase (18.0 U mg^−1^) were obtained from Toyobo Co. Uric acid and xanthine were sourced from Alfa Aesar. Medical tapes (M-tapes, 3M 468 MP) were purchased from 3M. PI films were sourced from DuPont, and polyethylene terephthalate (PET) films from McMaster-Carr.

### Fabrication of the BMS^3^ patch

The BMS^3^ sensor patch was fabricated using inkjet printing and laser micromachining. An inkjet printer (DMP-2850, Fujifilm) was used to sequentially print silver (interconnects, connection pads, and reference electrode), carbon (IP, counter electrode, and working electrode), and gold (working electrodes, four layers) onto a PI substrate. The reference and working electrodes were further modified using electrochemical deposition (CHI760E) and drop-casting for selective electrochemical sensing. A 50-W CO_2_ laser cutter (Universal Laser System) was used to pattern M-tape (3M 468MP), Janus membrane, microchannel, and PET layers, which were then assembled onto the sensor patch for microfluidic sweat processing.

#### Fabrication of Janus membranes and microchannels

A hydrophobic silica nanoparticle solution was prepared by dispersing 140 mg of silica in a 47 vol % PFOTS/53 vol % toluene solution. The mixture was heated at 100°C while stirring at 300 rpm for over 5 hours. After the reaction, the solution was centrifuged at 7500 to 8000 rpm for 5 min, and the supernatant was discarded. The precipitate was washed by adding fresh toluene, sonicating for 20 min, and repeating the process three times. Last, the purified silica nanoparticles were redispersed in anhydrous ethanol (25 mg ml^−1^).

Janus membranes and masks were patterned on 50-μm-thick PET using a CO_2_ laser cutter. A silica nanoparticle solution (25 mg ml^−1^ in anhydrous ethanol) was applied by dip-coating for 0.5 s. After drying, a mask was placed over the Janus membrane, and the hydrophilic area was fabricated via O_2_ plasma etching (Plasma Etch PE-25, 10 to 20 cm^3^ min^−1^ O_2_, 100 W, and 150 to 200 mtorr) for 16 min.

The hierarchical microchannels were fabricated on a PI film using photolithography with negative photoresist (SU-8 2015 and SU-8 2050, MicroChem). First, a PI film (PI2611, HD MicroSystems) was spin-coated at 3000 rpm and baked at 350°C for 30 min. Before patterning each SU-8 layer, an adhesion promoter (MCC Primer 80/20, MicroChem) was applied by spin-coating at 3000 rpm, followed by baking at 115°C for 1.5 min to enhance adhesion. For the base layer, SU-8 2015 was spin-coated at 4000 rpm for 30 s, soft-baked at 95°C for 4 min, exposed to ultraviolet (UV) at 145.5 mJ cm^−2^, and then hard-baked at 95°C for 5 min. To form the low ribs, SU-8 2015 was spin-coated at 1500 rpm for 30 s, soft-baked at 95°C for 4.5 min, exposed to UV at 160.5 mJ cm^−2^, and hard-baked at 95°C for 5.5 min. For the high ribs, SU-8 2050 was spin-coated at 2000 rpm for 30 s, soft-baked at 65°C for 3 min and 95°C for 9 min, exposed to UV at 220.5 mJ cm^−2^, and hard-baked at 65°C for 2 min and 95°C for 5.5 min. All SU-8 layers were developed using an SU-8 developer (KAYAKU ADVANCED MATERIALS) and rinsed with isopropyl alcohol.

#### Fabrication of the electrochemical sensor array

Enzymatic amperometric biosensors were developed for uric acid, xanthine, and alcohol detection. The gold surface was cleaned via CV for two cycles (0.2 to 1.5 V, 100 mV s^−1^) in 0.1 M H_2_SO_4_ using an electrochemical workstation (CHI 760E, CH Instruments).

For uric acid and xanthine sensors, a PB mediator layer was deposited using via pulsed technique (0.02-s step time, 1500 cycles) in a fresh solution of 2.5 mM FeCl_3_, 2.5 mM K_3_[Fe(CN)_6_], 100 mM KCl, and 100 mM HCl. For the alcohol sensor, a thicker PB mediator layer was fabricated using CV (−0.2 to 0.6 V, 50 mV s^−1^, and 10 cycles) using the same PB solution.

To improve sensor stability, a NiHCF layer was then further deposited using the same conditions as PB: pulsed deposition for uric acid and xanthine sensors and CV (−0.2 to 0.8 V, 50 mV s^−1^) for the alcohol sensor, in a solution containing 0.5 mM NiCl_2_, 0.5 mM K_3_[Fe(CN)_6_], and 1 M KCl.

For uric acid and xanthine sensors, enzyme cocktails were prepared by dissolving uricase (2.5 mg ml^−1^) or xanthine oxidase (30 mg ml^−1^) in a BSA-GA solution. The BSA solution (10 mg ml^−1^) was prepared in PBS and mixed with 2.5% GA (diluted from a 25% stock) at a 99:1 volume ratio. For the alcohol sensor, AOx was used as received and mixed with chitosan (1 wt % in 0.1 M acetic acid) and BSA (10 mg ml^−1^ in PBS) at a volume ratio of 1:1:8.

Each enzymatic electrode was drop-casted with 1.0 μl of enzyme cocktail and dried overnight at 4°C. For the alcohol sensor, a PU diffusion-limiting membrane was formed by dissolving 3 wt % PU in THF containing 2 wt % DMF via ultrasonication.

To prepare the pH sensor, the inkjet-printed Au surface was first cleaned via CV for 10 cycles (−0.1 to 0.9 V, 100 mV s^−1^) in 0.5 M HCl. PANI was then electrodeposited via CV for 12 cycles (−0.2 to 1.0 V, 100 mV s^−1^) under 0.1 M aniline in 1 M HCl. To ensure the growth of PANI on the Au surface, additional 12 CV cycles were processed with fresh aniline solution (0.1 M aniline in 1 M HCl).

The Ag/AgCl reference electrode was prepared by drop-casting 0.3 μl of 0.1 M FeCl_3_ onto the inkjet-printed Ag electrode surface for 30 s, followed by rinsing with deionized water. One microliter of PVB reference cocktail (prepared by dissolving 79.1 mg of PVB and 50 mg of NaCl in 1 ml of methanol) was then applied on the electrode, followed by overnight drying and drop-casted.

#### IP gel fabrication

Both anode and cathode IP gels were prepared by dissolving 3% (w/w) agarose in deionized water at 95°C under constant stirring until homogeneous. The solution was cooled to 84°C before adding 1% (w/w) carbachol (anode) and 1% (w/w) NaCl (cathode), followed by thorough mixing and pouring into reservoirs.

### In vitro sensor characterization and validation

Electrochemical performance of the enzymatic biosensors was evaluated using CV and amperometry (CHI 1430, CH Instruments). Analyte solutions were all prepared in 1× PBS (pH 9.4) unless specified: uric acid (0 to 30 μM), xanthine (0 to 20 μM), and ethanol (0 to 20 mM). Amperometric measurements were conducted at 0 V. For pH characterization, open-circuit potential (OCP) measurements were performed in McIlvaine’s buffers (pH 4 to 8) and NaOH-mediated McIlvaine buffer with a pH of 9. Temperature effects were assessed using a ceramic hot plate (Thermo Fisher Scientific). Potentiometric and chronoamperometric responses were recorded at 1-s intervals, except for long-term monitoring, which used a 5-s interval to reduce data overload.

The performance of the enzymatic biosensors was validated by comparing sweat analyte measurements with those obtained from commercial assay kits. To ensure the sensor’s accuracy across the relevant concentration range, sweat samples were collected from subjects under varying intake conditions, including fasting, and postconsumption of tuna, soft drinks, or beer, providing a broad spectrum of analyte concentrations for evaluation.

### In vitro and in vivo evaluation of microfluidics for enhanced sweat sampling

In vitro flow tests were conducted using a syringe pump (78-01001, Thermo Fisher Scientific) at 0.1 μl min^−1^ to assess the dynamic response of the enzymatic sensors in the BMS^3^ compared to a control system lacking the Janus membrane and microchannels.

For in vivo sweat sampling test, a blue dye droplet was placed in the sweat reservoir, and BMS^3^ was attached to the forearm of the subject. Sweat induction was performed using the macroduct sweat collection system with carbachol hydrogel and a controlled current applied for 5 min. Sweat collection was analyzed at 8, 24, and 48 hours post-IP to assess the microfluidic module’s capability for sweat harvesting and sampling over an extended period.

### Electronic system design and characterization

The electronic system of the BMS^3^ consists of four main blocks. The power management block includes a boost converter (TPS61096, Texas Instruments) that steps up a 3.7-V LiPo battery to an 18-V high compliance voltage for IP and a voltage regulator (LD39050, STMicroelectronics) that supplies a stable 3.3 V for the microcontroller unit (MCU) and sensor interface. The data processing and wireless communication block features an STM32WB5M microcontroller with integrated Bluetooth Low Energy (BLE) (STMicroelectronics). The electrochemical instrumentation block comprises a quad op-amp (TLV9004, Texas Instruments) for three-channel amperometry, two instrumentation amplifiers (INA333, Texas Instruments) for two-channel potentiometry, a voltage divider for temperature measurement, and a switch matrix (TMUX1112, Texas Instruments) that connects sensor electrodes to the sensor circuit. The IP block uses a current mirror built from a dual transistor array (BCV62, Nexperia), a current shunt monitor (INA285, Texas Instruments) for precise current tracking, and a high-voltage switch (MAX4648, Analog Devices) for safely delivering current to the electrodes.

### Biocompatibility and cytocompatibility of the BMS^3^ patch

We assessed the biocompatibility of the device extract in vitro using the murine fibroblast cell line CCL-1 (L929), a standard model for cytotoxicity evaluation. We cultured cells in Eagle’s minimum essential medium (EMEM) supplemented with 10% fetal bovine serum (FBS) and maintained at 37°C in a humidified incubator with 5% CO_2_. Subculturing was performed once cultures reached ~70% confluency, and only cells at passages 3 to 5 were used in experiments to ensure consistency and cell health. We prepared device extracts by soaking the device in cell culture medium and then applied to L929 monolayers for 24 hours. To assess cytotoxic response, we conducted two complementary assays. We measured metabolic activity using the MTT assay, where mitochondrial dehydrogenases in viable cells reduce MTT to insoluble formazan, which was quantified by absorbance at 570 nm. In parallel, we evaluated cell membrane integrity and viability using a live/dead fluorescence staining kit (Calcein AM/Ethidium Homodimer-1), which distinguishes live cells (green fluorescence) from dead cells (red fluorescence). Together, these assays provided both quantitative and visual confirmation of the material’s cytocompatibility.

### Evaluation of BMS^3^ in human participants

We conducted validation and evaluation of the BMS^3^ in compliance with ethical regulations under a protocol (ID 24-0892) approved by the Institutional Review Board at the California Institute of Technology (Caltech). Human subjects aged 18 to 65 were recruited from the Caltech campus and surrounding areas via advertisements, word of mouth, and email distribution. Patients with gout were additionally required to have serum urate levels exceeding 6 mg dl^−1^ for women and 7 mg dl^−1^ for men, regardless of current or recent urate lowering medications, (such as allopurinol). Written informed consent was obtained from all participants before any study procedures. Before sensor application, all participants cleaned their skin using water and alcohol swabs.

During on-body trials, all participants underwent one IP sweat stimulation section, and multimodal physiochemical signals—including temperature, pH, and sweat biomarkers—were wirelessly transmitted via BLE. For multiday evaluations, participants performed various daily activities over a 4-hour period, enabling long-term tracking of sweat biomarker dynamics and real-world usability of BMS^3^. To assess dietary influence on metabolic biomarkers, participants consumed either 85 g of tuna with a soft drink containing 39 g of sugar or 85 g of tuna with one bottle of beer, followed by 2 hours of continuous metabolic monitoring. For the allopurinol influence test, gout patient participants received their prescribed dose of allopurinol, and their biochemical response was monitored over a 2-hour period to assess changes in uric acid and xanthine levels. For the blood/sweat correlation study, patient participants consumed a high-purine meal (sardines and soda) while wearing the BMS^3^ patch. Participants undergoing treatment also took allopurinol. Uric acid levels in sweat were continuously monitored using the BMS^3^ patch, whereas serum uric acid levels were measured from blood samples using a commercial assay kit (Amplex Red Uric Acid/Uricase Assay Kit, Invitrogen).

## References

[1] W. Gao, S. Emaminejad, H. Y. Y. Nyein, S. Challa, K. Chen, A. Peck, H. M. Fahad, H. Ota, H. Shiraki, D. Kiriya, D. H. Lien, G. A. Brooks, R. W. Davis, A. Javey, Fully integrated wearable sensor arrays for multiplexed in situ perspiration analysis. Nature 529, 509–514 (2016). 26819044 10.1038/nature16521 PMC4996079

[2] J. Min, J. Tu, C. Xu, H. Lukas, S. Shin, Y. Yang, S. A. Solomon, D. Mukasa, W. Gao, Skin-interfaced wearable sweat sensors for precision medicine. Chem. Rev. 123, 5049–5138 (2023). 36971504 10.1021/acs.chemrev.2c00823 PMC10406569

[3] J. Heikenfeld, A. Jajack, B. Feldman, S. W. Granger, S. Gaitonde, G. Begtrup, B. A. Katchman, Accessing analytes in biofluids for peripheral biochemical monitoring. Nat. Biotechnol. 37, 407–419 (2019). 30804536 10.1038/s41587-019-0040-3

[4] J. Kim, A. S. Campbell, B. E.-F. de Ávila, J. Wang, Wearable biosensors for healthcare monitoring. Nat. Biotechnol. 37, 389–406 (2019). 30804534 10.1038/s41587-019-0045-y PMC8183422

[5] M. Bariya, H. Y. Y. Nyein, A. Javey, Wearable sweat sensors. Nat. Electron. 1, 160–171 (2018).

[6] N. Brasier, J. Wang, W. Gao, J. R. Sempionatto, C. Dincer, H. C. Ates, F. Güder, S. Olenik, I. Schauwecker, D. Schaffarczyk, E. Vayena, N. Ritz, M. Weisser, S. Mtenga, R. Ghaffari, J. A. Rogers, J. Goldhahn, Applied body-fluid analysis by wearable devices. Nature 636, 57–68 (2024). 39633192 10.1038/s41586-024-08249-4 PMC12007731

[7] D. S. Yang, R. Ghaffari, J. A. Rogers, Sweat as a diagnostic biofluid. Science 379, 760–761 (2023). 36821680 10.1126/science.abq5916

[8] Y. Yang, W. Gao, Wearable and flexible electronics for continuous molecular monitoring. Chem. Soc. Rev. 48, 1465–1491 (2019). 29611861 10.1039/c7cs00730b

[9] S. Cho, S. M. Shaban, R. Song, H. Zhang, D. Yang, M.-J. Kim, Y. Xiong, X. Li, K. Madsen, S. Wapnick, S. Zhang, Z. Chen, J. Kim, G. Guinto, M. Li, M. Lee, R. F. Nuxoll, S. Shajari, J. Wang, S. Son, J. Shin, A. J. Aranyosi, D. E. Wright, T. Kim, R. Ghaffari, Y. Huang, D.-H. Kim, J. A. Rogers, A skin-interfaced microfluidic platform supports dynamic sweat biochemical analysis during human exercise. Sci. Transl. Med. 16, eado5366 (2024). 39231240 10.1126/scitranslmed.ado5366

[10] L. B. Baker, J. B. Model, K. A. Barnes, M. L. Anderson, S. P. Lee, K. A. Lee, S. D. Brown, A. J. Reimel, T. J. Roberts, R. P. Nuccio, J. L. Bonsignore, C. T. Ungaro, J. M. Carter, W. Li, M. S. Seib, J. T. Reeder, A. J. Aranyosi, J. A. Rogers, R. Ghaffari, Skin-interfaced microfluidic system with personalized sweating rate and sweat chloride analytics for sports science applications. Sci. Adv. 6, eabe3929 (2020). 33310859 10.1126/sciadv.abe3929 PMC7732194

[11] J. Cai, M. Cao, J. Bai, M. Sun, C. Ma, M. Y. Emran, A. Kotb, X. Bo, M. Zhou, Flexible epidermal wearable sensor for Athlete’s sweat biomarkers monitoring. Talanta 282, 126986 (2025). 39383716 10.1016/j.talanta.2024.126986

[12] M. Wang, Y. Yang, J. Min, Y. Song, J. Tu, D. Mukasa, C. Ye, C. Xu, N. Heflin, J. S. McCune, T. K. Hsiai, Z. Li, W. Gao, A wearable electrochemical biosensor for the monitoring of metabolites and nutrients. Nat. Biomed. Eng 6, 1225–1235 (2022). 35970928 10.1038/s41551-022-00916-z PMC10432133

[13] J. Kim, Y. Wu, H. Luan, D. S. Yang, D. Cho, S. S. Kwak, S. Liu, H. Ryu, R. Ghaffari, J. A. Rogers, A skin-interfaced, miniaturized microfluidic analysis and delivery system for colorimetric measurements of nutrients in sweat and supply of vitamins through the skin. Adv. Sci. 9, e2103331 (2022). 10.1002/advs.202103331 PMC8805554 34747140

[14] Y. Yang, Y. Song, X. Bo, J. Min, O. S. Pak, L. Zhu, M. Wang, J. Tu, A. Kogan, H. Zhang, T. K. Hsiai, Z. Li, W. Gao, A laser-engraved wearable sensor for sensitive detection of uric acid and tyrosine in sweat. Nat. Biotechnol. 38, 217–224 (2020). 31768044 10.1038/s41587-019-0321-x

[15] J. R. Sempionatto, A. A. Khorshed, A. Ahmed, A. N. De Loyola e Silva, A. Barfidokht, L. Yin, K. Y. Goud, M. A. Mohamed, E. Bailey, J. May, C. Aebischer, C. Chatelle, J. Wang, Epidermal enzymatic biosensors for sweat vitamin C: Toward personalized nutrition. ACS Sens. 5, 1804–1813 (2020). 32366089 10.1021/acssensors.0c00604

[16] D. Mukasa, M. Wang, J. Min, Y. Yang, S. A. Solomon, H. Han, C. Ye, W. Gao, A computationally assisted approach for designing wearable biosensors toward non-invasive personalized molecular analysis. Adv. Mater. 35, e2212161 (2023). 37159949 10.1002/adma.202212161 PMC10529901

[17] J. Tu, J. Min, Y. Song, C. Xu, J. Li, J. Moore, J. Hanson, E. Hu, T. Parimon, T.-Y. Wang, E. Davoodi, T.-F. Chou, P. Chen, J. J. Hsu, H. B. Rossiter, W. Gao, A wireless patch for the monitoring of C-reactive protein in sweat. Nat. Biomed. Eng 7, 1293–1306 (2023). 37349389 10.1038/s41551-023-01059-5 PMC10592261

[18] T. R. Ray, M. Ivanovic, P. M. Curtis, D. Franklin, K. Guventurk, W. J. Jeang, J. Chafetz, H. Gaertner, G. Young, S. Rebollo, J. B. Model, S. P. Lee, J. Ciraldo, J. T. Reeder, A. Hourlier-Fargette, A. J. Bandodkar, J. Choi, A. J. Aranyosi, R. Ghaffari, S. A. McColley, S. Haymond, J. A. Rogers, Soft, skin-interfaced sweat stickers for cystic fibrosis diagnosis and management. Sci. Transl. Med. 13, eabd8109 (2021). 33790027 10.1126/scitranslmed.abd8109 PMC8351625

[19] A. Veronica, Y. Li, Y. Li, I.-M. Hsing, H. Y. Y. Nyein, Dermal-fluid-enabled detection platforms for non-invasive ambulatory monitoring. Sens. Diagn. 2, 1335–1359 (2023).

[20] R. M. Torrente-Rodríguez, J. Tu, Y. Yang, J. Min, M. Wang, Y. Song, Y. Yu, C. Xu, C. Ye, W. W. IsHak, W. Gao, Investigation of cortisol dynamics in human sweat using a graphene-based wireless mHealth system. Matter 2, 921–937 (2020). 32266329 10.1016/j.matt.2020.01.021 PMC7138219

[21] C. Xu, Y. Song, J. R. Sempionatto, S. A. Solomon, Y. Yu, H. Y. Y. Nyein, R. Y. Tay, J. Li, W. Heng, J. Min, A. Lao, T. K. Hsiai, J. A. Sumner, W. Gao, A physicochemical-sensing electronic skin for stress response monitoring. Nat. Electron. 7, 168–179 (2024). 38433871 10.1038/s41928-023-01116-6 PMC10906959

[22] B. Wang, C. Zhao, Z. Wang, K.-A. Yang, X. Cheng, W. Liu, W. Yu, S. Lin, Y. Zhao, K. M. Cheung, H. Lin, H. Hojaiji, P. S. Weiss, M. N. Stojanović, A. J. Tomiyama, A. M. Andrews, S. Emaminejad, Wearable aptamer-field-effect transistor sensing system for noninvasive cortisol monitoring. Sci. Adv. 8, eabk0967 (2022). 34985954 10.1126/sciadv.abk0967 PMC8730602

[23] O. Parlak, S. T. Keene, A. Marais, V. F. Curto, A. Salleo, Molecularly selective nanoporous membrane-based wearable organic electrochemical device for noninvasive cortisol sensing. Sci. Adv. 4, eaar2904 (2018). 30035216 10.1126/sciadv.aar2904 PMC6054510

[24] W. Tang, L. Yin, J. R. Sempionatto, J. Moon, H. Teymourian, J. Wang, Touch-based stressless cortisol sensing. Adv. Mater. 33, e2008465 (2021). 33786887 10.1002/adma.202008465

[25] C. Ye, M. Wang, J. Min, R. Y. Tay, H. Lukas, J. R. Sempionatto, J. Li, C. Xu, W. Gao, A wearable aptamer nanobiosensor for non-invasive female hormone monitoring. Nat. Nanotechnol. 19, 330–337 (2024). 37770648 10.1038/s41565-023-01513-0 PMC10954395

[26] J. Moon, H. Teymourian, E. De La Paz, J. R. Sempionatto, K. Mahato, T. Sonsa-ard, N. Huang, K. Longardner, I. Litvan, J. Wang, Non-invasive sweat-based tracking of L-dopa pharmacokinetic profiles following an oral tablet administration. Angew. Chem. Int. Ed. Engl. 60, 19074–19078 (2021). 34145703 10.1002/anie.202106674 PMC8373796

[27] L.-C. Tai, W. Gao, M. Chao, M. Bariya, Q. P. Ngo, Z. Shahpar, H. Y. Y. Nyein, H. Park, J. Sun, Y. Jung, E. Wu, H. M. Fahad, D.-H. Lien, H. Ota, G. Cho, A. Javey, Methylxanthine drug monitoring with wearable sweat sensors. Adv. Mater. 30, e1707442 (2018). 29663538 10.1002/adma.201707442

[28] S. Lin, W. Yu, B. Wang, Y. Zhao, K. En, J. Zhu, X. Cheng, C. Zhou, H. Lin, Z. Wang, H. Hojaiji, C. Yeung, C. Milla, R. W. Davis, S. Emaminejad, Noninvasive wearable electroactive pharmaceutical monitoring for personalized therapeutics. Proc. Natl. Acad. Sci. U.S.A. 117, 19017–19025 (2020). 32719130 10.1073/pnas.2009979117 PMC7431025

[29] C. Liu, T. Xu, D. Wang, X. Zhang, The role of sampling in wearable sweat sensors. Talanta 212, 120801 (2020). 32113563 10.1016/j.talanta.2020.120801

[30] H. Y. Y. Nyein, M. Bariya, B. Tran, C. H. Ahn, B. J. Brown, W. Ji, N. Davis, A. Javey, A wearable patch for continuous analysis of thermoregulatory sweat at rest. Nat. Commun. 12, 1823 (2021). 33758197 10.1038/s41467-021-22109-z PMC7987967

[31] R. T. Arwani, S. C. L. Tan, A. Sundarapandi, W. P. Goh, Y. Liu, F. Y. Leong, W. Yang, X. T. Zheng, Y. Yu, C. Jiang, Y. C. Ang, L. Kong, S. L. Teo, P. Chen, X. Su, H. Li, Z. Liu, X. Chen, L. Yang, Y. Liu, Stretchable ionic–electronic bilayer hydrogel electronics enable in situ detection of solid-state epidermal biomarkers. Nat. Mater. 23, 1115–1122 (2024). 38867019 10.1038/s41563-024-01918-9

[32] J. R. Sempionatto, J.-M. Moon, J. Wang, Touch-based fingertip blood-free reliable glucose monitoring: Personalized data processing for predicting blood glucose concentrations. ACS Sens. 6, 1875–1883 (2021). 33872007 10.1021/acssensors.1c00139

[33] J. Kim, I. Jeerapan, S. Imani, T. N. Cho, A. Bandodkar, S. Cinti, P. P. Mercier, J. Wang, Noninvasive alcohol monitoring using a wearable tattoo-based iontophoretic-biosensing system. ACS Sens. 1, 1011–1019 (2016).

[34] S. Emaminejad, W. Gao, E. Wu, Z. A. Davies, H. Y. Y. Nyein, S. Challa, S. P. Ryan, H. M. Fahad, K. Chen, Z. Shahpar, S. Talebi, C. Milla, A. Javey, R. W. Davis, Autonomous sweat extraction and analysis applied to cystic fibrosis and glucose monitoring using a fully integrated wearable platform. Proc. Natl. Acad. Sci. U.S.A. 114, 4625–4630 (2017). 28416667 10.1073/pnas.1701740114 PMC5422810

[35] Z. Sonner, E. Wilder, T. Gaillard, G. Kasting, J. Heikenfeld, Integrated sudomotor axon reflex sweat stimulation for continuous sweat analyte analysis with individuals at rest. Lab Chip 17, 2550–2560 (2017). 28675233 10.1039/c7lc00364a

[36] J. Min, S. Demchyshyn, J. R. Sempionatto, Y. Song, B. Hailegnaw, C. Xu, Y. Yang, S. Solomon, C. Putz, L. E. Lehner, J. F. Schwarz, C. Schwarzinger, M. C. Scharber, E. Shirzaei Sani, M. Kaltenbrunner, W. Gao, An autonomous wearable biosensor powered by a perovskite solar cell. Nat. Electron. 6, 630–641 (2023). 38465017 10.1038/s41928-023-00996-y PMC10923186

[37] Y. Wang, M. Deng, B. Deng, L. Ye, X. Fei, Z. Huang, Study on the diagnosis of gout with xanthine and hypoxanthine. J. Clin. Lab. Anal. 33, e22868 (2019). 30803031 10.1002/jcla.22868 PMC6595306

[38] K. L. Rock, H. Kataoka, J.-J. Lai, Uric acid as a danger signal in gout and its comorbidities. Nat. Rev. Rheumatol. 9, 13–23 (2013). 22945591 10.1038/nrrheum.2012.143 PMC3648987

[39] H. K. Choi, K. Atkinson, E. W. Karlson, W. Willett, G. Curhan, Alcohol intake and risk of incident gout in men: A prospective study. Lancet 363, 1277–1281 (2004). 15094272 10.1016/S0140-6736(04)16000-5

[40] T. Yamamoto, Y. Moriwaki, S. Takahashi, Effect of ethanol on metabolism of purine bases (hypoxanthine, xanthine, and uric acid). Clin. Chim. Acta 356, 35–57 (2005). 15936302 10.1016/j.cccn.2005.01.024

[41] Z. Dai, M. Lei, S. Ding, Q. Zhou, B. Ji, M. Wang, B. Zhou, Durable superhydrophobic surface in wearable sensors: From nature to application. Exploration 4, 20230046 (2024). 38855620 10.1002/EXP.20230046 PMC11022629

[42] Y. Liu, R. Qu, W. Zhang, X. Li, Y. Wei, L. Feng, Lotus- and mussel-inspired PDA–PET/PTFE Janus membrane: Toward integrated separation of light and heavy oils from water. ACS Appl. Mater. Interfaces 11, 20545–20556 (2019). 31082194 10.1021/acsami.9b04775

[43] X. He, T. Xu, Z. Gu, W. Gao, L.-P. Xu, T. Pan, X. Zhang, Flexible and superwettable bands as a platform toward sweat sampling and sensing. Anal. Chem. 91, 4296–4300 (2019). 30880389 10.1021/acs.analchem.8b05875

[44] Y. Wang, W. Cai, Y. Zhang, J. Ji, H. Zheng, D. Yan, X. Liu, Superhydrophobic wearable sensor: Fabrication, application, and perspective. Discov. Nano 19, 176 (2024). 39514134 10.1186/s11671-024-04138-x PMC11549076

[45] X. He, S. Yang, Q. Pei, Y. Song, C. Liu, T. Xu, X. Zhang, Integrated smart janus textile bands for self-pumping sweat sampling and analysis. ACS Sens. 5, 1548–1554 (2020). 32466645 10.1021/acssensors.0c00563

[46] T. Wu, P. Yang, X. Xie, X. Cao, Y. Deng, X. Ding, Z. Zhang, Bio-inspired hierarchical wearable patch for fast sweat collection. Biosens. Bioelectron. 260, 116430 (2024). 38815465 10.1016/j.bios.2024.116430

[47] H. Chen, T. Ran, Y. Gan, J. Zhou, Y. Zhang, L. Zhang, D. Zhang, L. Jiang, Ultrafast water harvesting and transport in hierarchical microchannels. Nat. Mater. 17, 935–942 (2018). 30250072 10.1038/s41563-018-0171-9

[48] J. George, A. D. Struthers, Role of urate, xanthine oxidase and the effects of allopurinol in vascular oxidative stress. Vasc. Health Risk Manag. 5, 265–272 (2009). 19436671 10.2147/vhrm.s4265 PMC2672460

[49] D. F. B. Wright, L. K. Stamp, T. R. Merriman, M. L. Barclay, S. B. Duffull, N. H. G. Holford, The population pharmacokinetics of allopurinol and oxypurinol in patients with gout. Eur. J. Clin. Pharmacol. 69, 1411–1421 (2013). 23475133 10.1007/s00228-013-1478-8

[50] R. O. Day, G. G. Graham, M. Hicks, A. J. McLachlan, S. L. Stocker, K. M. Williams, Clinical pharmacokinetics and pharmacodynamics of allopurinol and oxypurinol. Clin. Pharmacokinet. 46, 623–644 (2007). 17655371 10.2165/00003088-200746080-00001

