# Supplementary material for: A bioinspired microfluidic wearable sensor for multiday sweat sampling, transport, and metabolic analysis
Source: Sci Adv. 2025 Aug 13;11(33):eadw9024. doi: 10.1126/sciadv.adw9024 (PMC12346344; doi:10.1126/sciadv.adw9024)
Supplement: Supplementary file 1 — Figs. S1 to S29 Legends for movies S1 to S3 [file sciadv.adw9024_sm.pdf]

Science Advances
AAAS

Supplementary Materials for

A bioinspired microfluidic wearable sensor for multiday sweat sampling, transport, and metabolic analysis

Soyoung Shin *et al.*

Corresponding author: Wei Gao, weigao@caltech.edu

Sci. Adv. **11**, eadw9024 (2025)
DOI: 10.1126/sciadv.adw9024

The PDF file includes:

Figs. S1 to S29
Legends for movies S1 to S3

Other Supplementary Material for this manuscript includes the following:

Movies S1 to S3

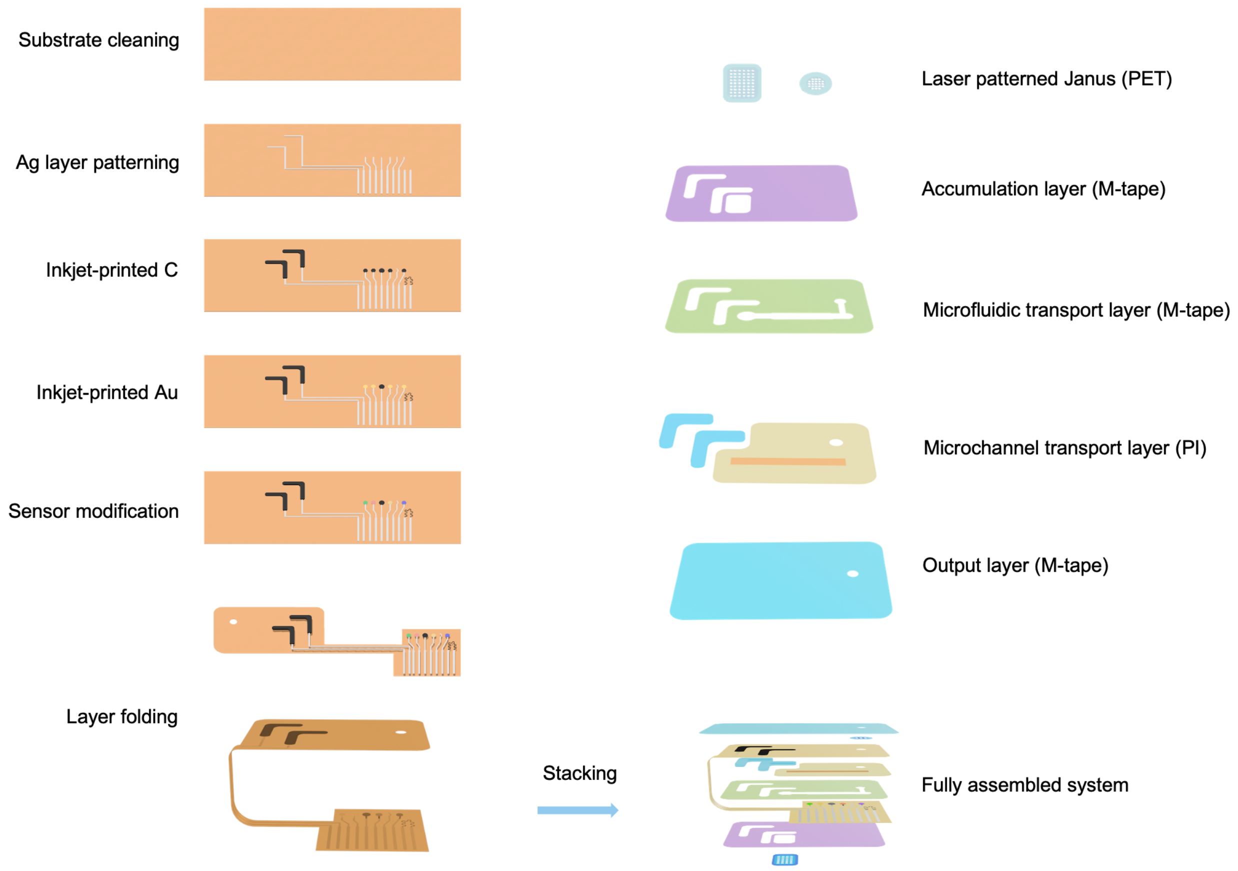

Fig. S1. Fabrication steps of the BMS³ sensor patch.

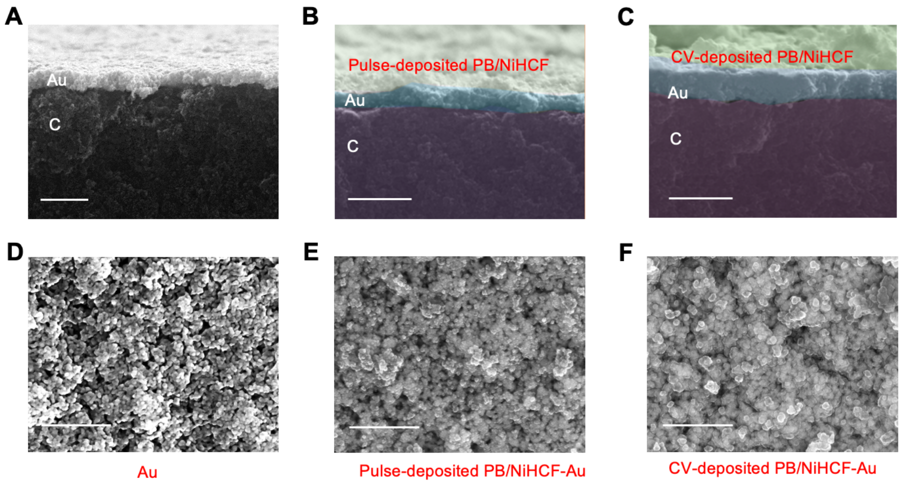

Fig. S2. Scanning electron microscope (SEM) images of the layered structure of the sensing electrodes. (**A** to **C**) SEM images show the surface morphology of the electrodes, with corresponding high-resolution cross-sectional features for printed C and Au layers (**A**), mediator (PB) and stabilizer (NiHCF) layers for the uric acid/xanthine sensor (**B**) and for the alcohol sensor (**C**). (**D** to **F**) Top-view SEM images displaying the printed C and Au (**D**), mediator (PB) and stabilizer (NiHCF) layers for the xanthine/uric acid sensor (**E**) and for the alcohol sensor (**F**). Scale bar, 1 µm.

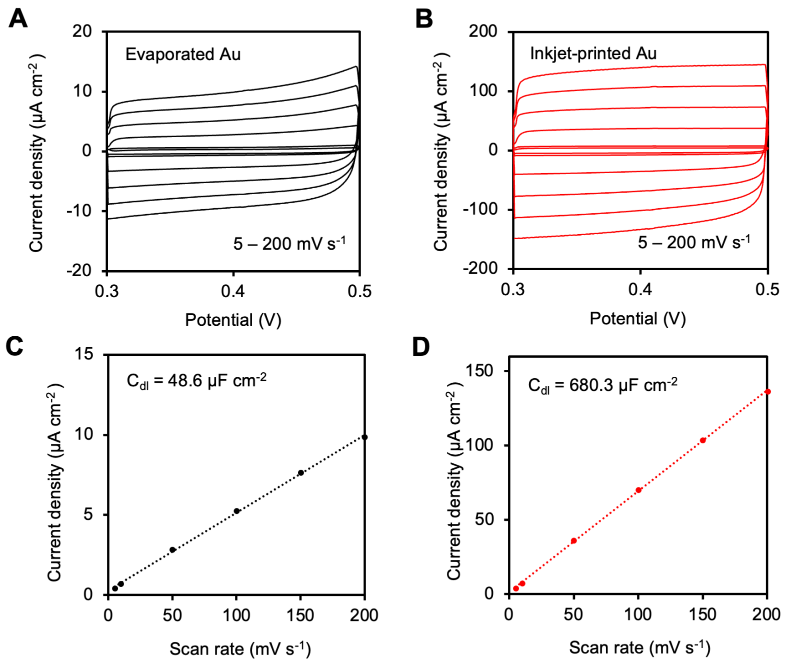

Fig. S3. Electrochemical characterization of the Au electrodes. (**A** and **B**) Cyclic voltammetry (CV) conducted on evaporated Au (**A**) and inkjet-printed Au (**B**) electrodes at various scan rates (5, 10, 50, 100, 150, 200 mV s^{-1}) within a potential range of 0.3–0.5 V in 1× PBS (pH 7.4). (**C** and **D**) The corresponding calibration plots of oxidation peak height current density at 0.4 V versus scan rate for evaporated Au (**C**) and inkjet-printed Au (**D**), with dotted lines indicating linear trendlines. Electrochemical double-layer capacitance (C_{dl}) was used to estimate the electrochemical surface area (ECSA) of the electrodes.

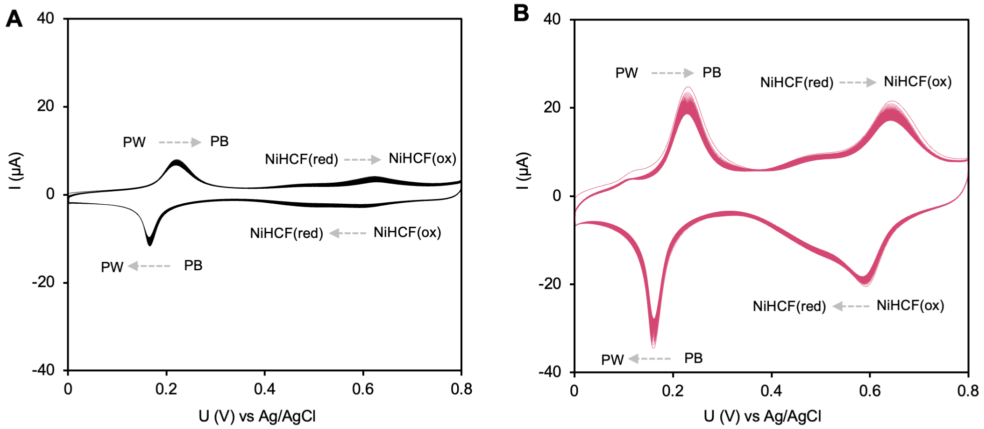

Fig. S4. Electrochemical stability of mediator and stabilizer. (**A** and **B**) 100 cycles of CV in 0.1M KCl for xanthine/uric acid sensor (**A**) and alcohol sensor (**B**), with an applied potential range of 0 to 0.8 V to evaluate the stability of the mediator Prussian Blue (PB) and stabilizer NiHCF. Scan rate, 50 mV s^{-1}. PW, Prussian white; PB, Prussian blue; NiHCF(red), reduced form of NiHCF; NiHCF(ox), oxidized form of NiHCF.

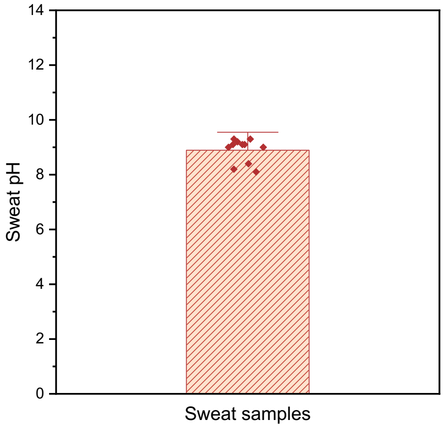

Fig. S5. pH of carbachol induced sweat. Error bars represent s.d. of the mean from 11 subjects.

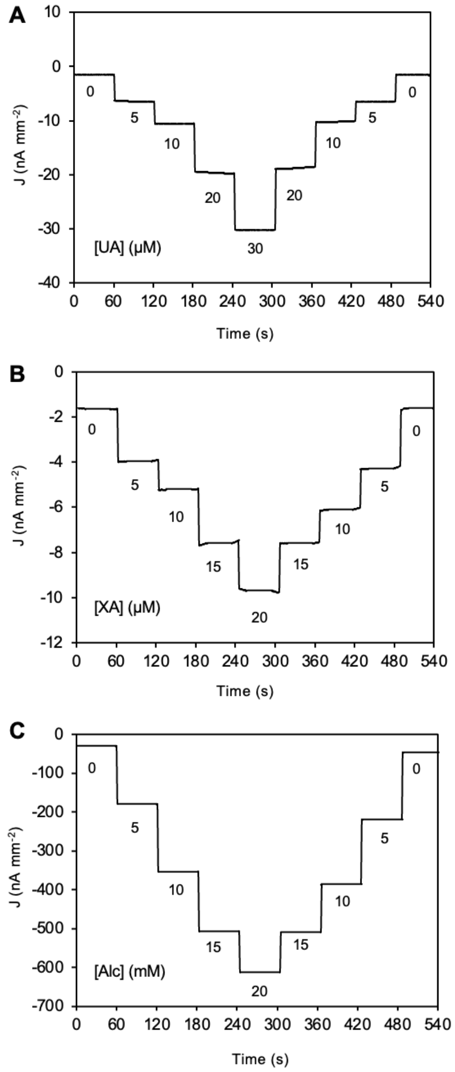

Fig. S6. Reversibility characterization of enzymatic biosensors. (**A** to **C**) Amperometric responses of the xanthine (**A**), uric acid (**B**), and alcohol (**C**) sensors with varying concentrations of target analyte. Sensor responses were recorded by sequentially increasing (forward) and decreasing (backward) analyte concentrations.

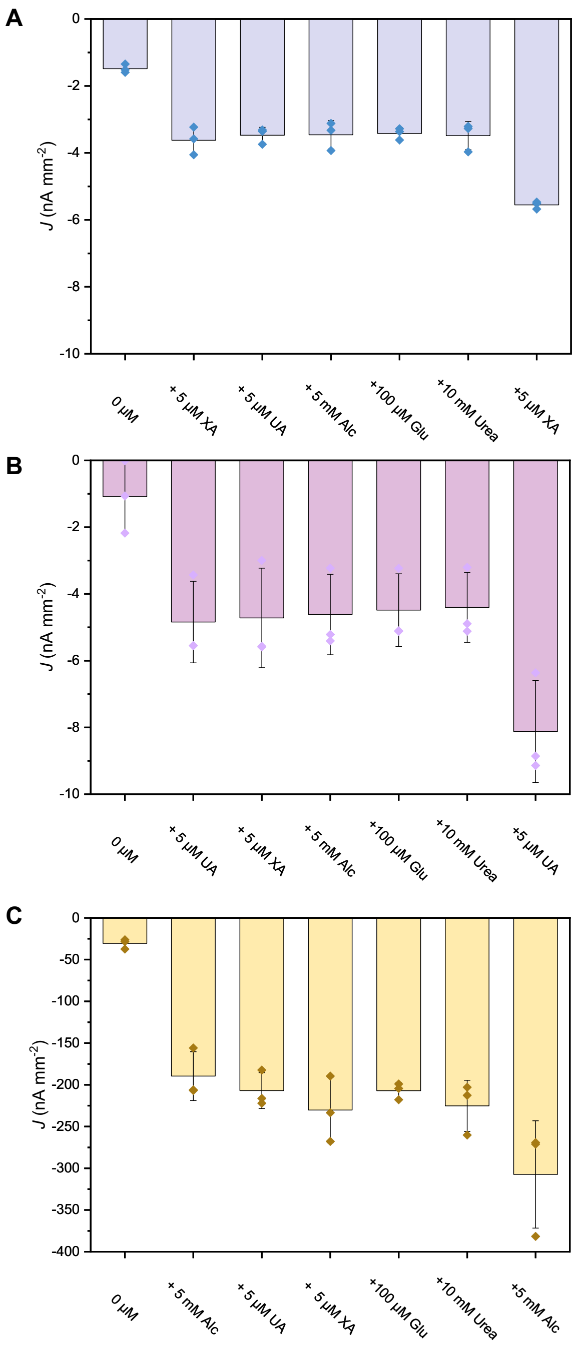

Fig. S7. Selectivity of enzymatic biosensors. (**A** to **C**) Selectivity test of xanthine (**A**), uric acid (**B**), and alcohol (**C**) sensors. Glu, glucose. Error bars represent S.D. of the mean from 3 sensors.

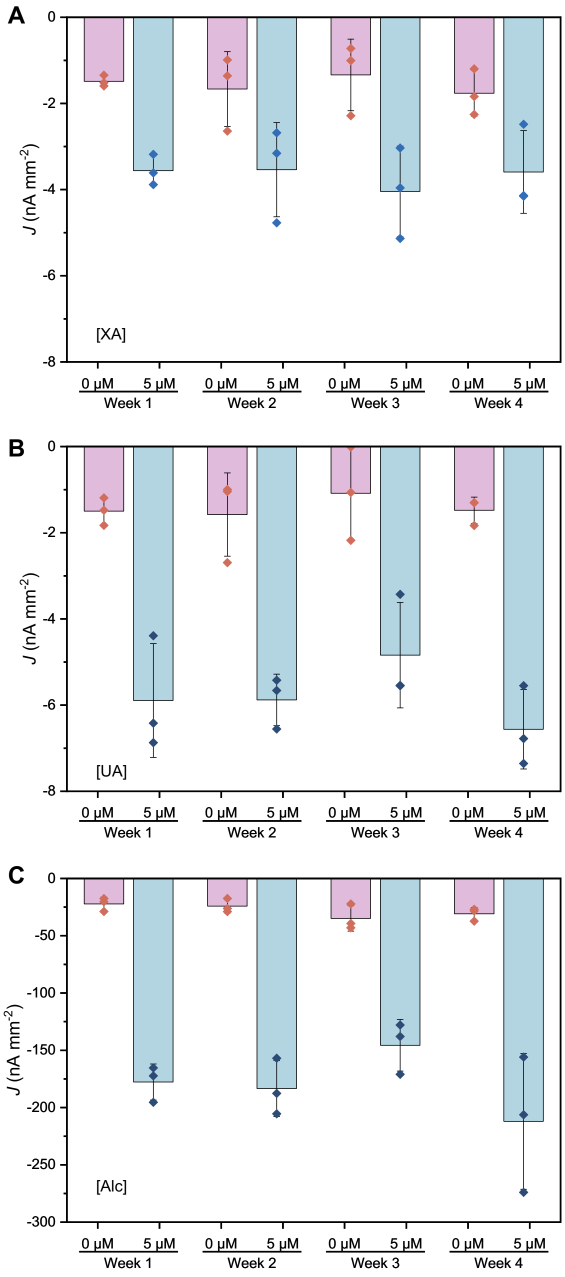

Fig. S8. Shelf stability of enzymatic biosensors. (**A** to **C**) Amperometric responses of xanthine (**A**), uric acid (**B**), and alcohol (**C**) sensors after storage in the refrigerator for 1–4 weeks. Error bars represent S.D. of the mean from 3 sensors.

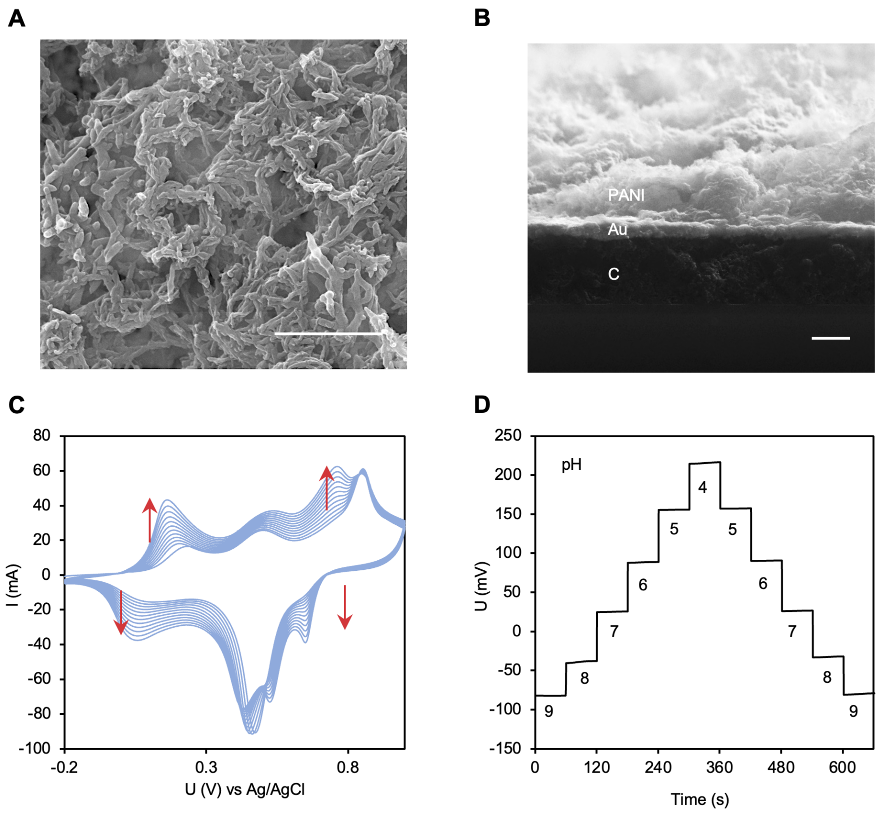

Fig. S9. Characterizations of pH sensors. (**A** and **B**) Top view (**A**) and cross-sectional view (**B**) SEM images of the pH-sensitive PANI film. Scale bars, 1 µm. (**C**) CV of PANI deposition. Scan rate, 100 mV s^{-1}; 12 cycles. (**D**) Potentiometric response of a PANI-based pH sensor tested from pH 9 to 4 and vice versa.

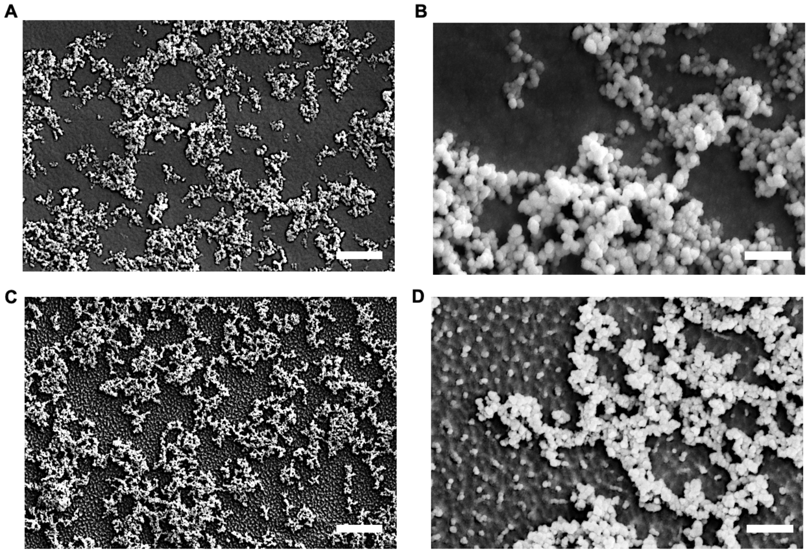

Fig. S10. SEM images of the silica nanoparticle coatings. (**A** to **D**) The untreated (**A** and **B**) and O_2 plasma treated (**C** and **D**) silica nanoparticle coating. Scale bars, 1 μm for **A** and **C**, 100 nm for **B** and **D**.

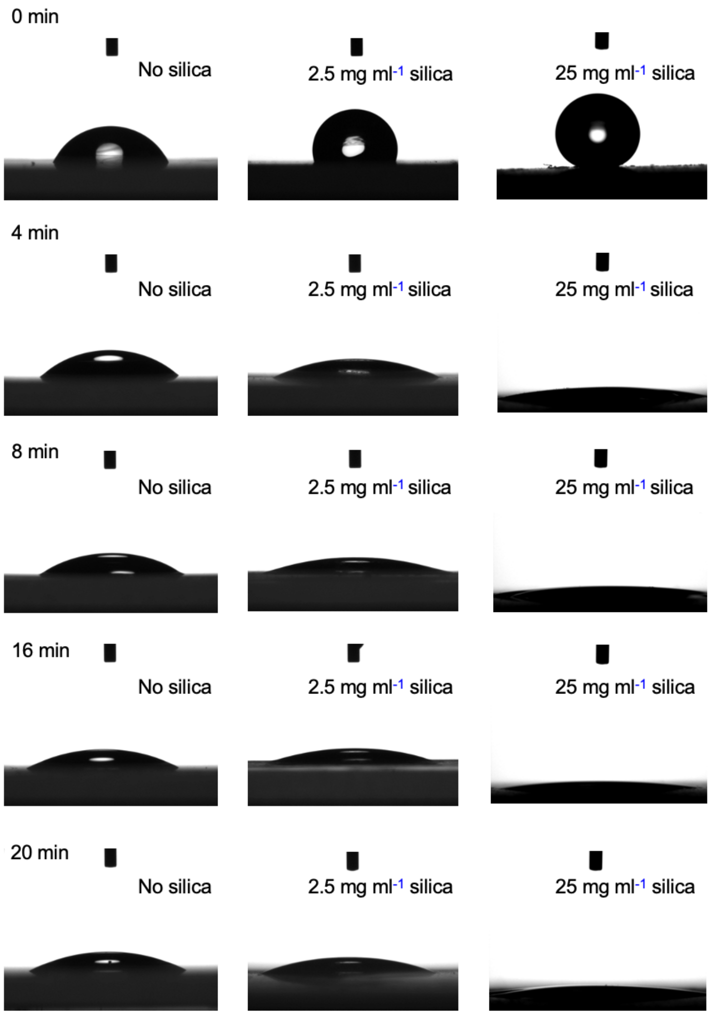

Fig. S11. Contact angles of the silica coated surfaces under varying O_2 plasma etching time and silica concentrations. 50 μm PET were coated with different concentration of silica (0, 2.5, and 25 mg ml^{-1}) to form hydrophobic surface. O_2 plasma was processed in different length (4, 8, 16, and 20 min) on silica-coated surface to form hydrophilic surface.

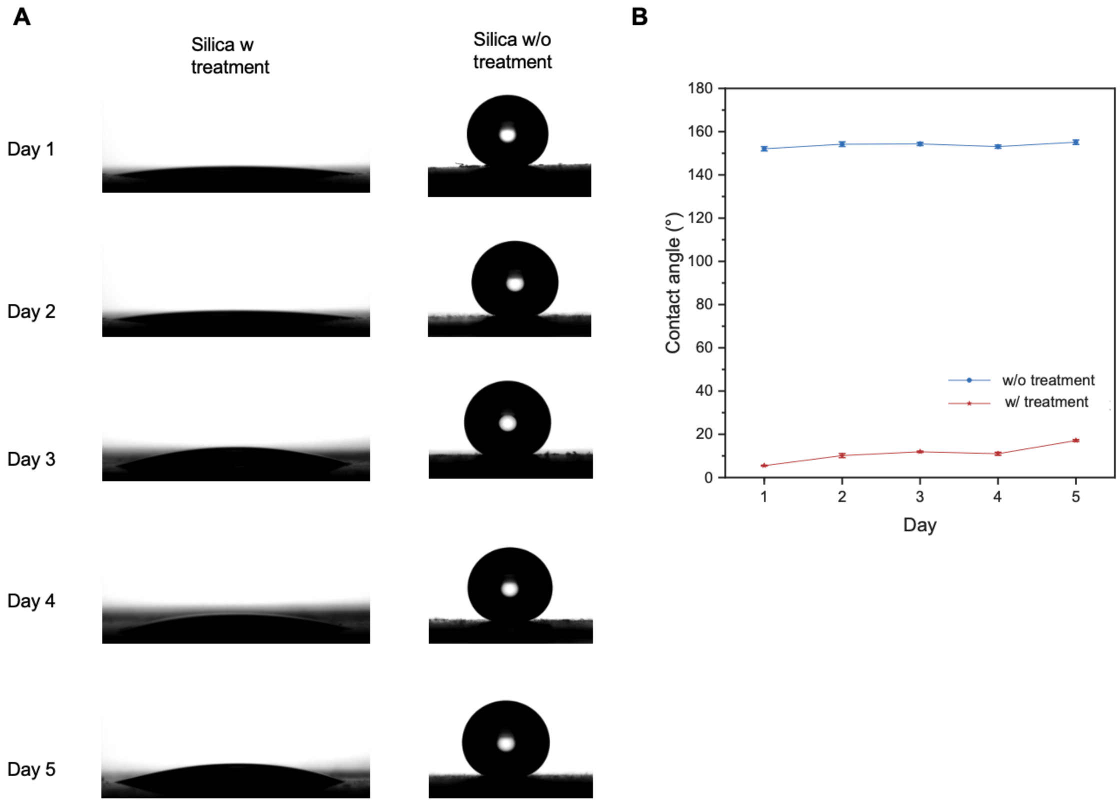

Fig. S12. Wettability and shelf stability of the Janus membrane. (**A** and **B**) Wettability (**A**) and shelf stability (**B**) tests for both superhydrophilic (silica with O_2 treatment) and superhydrophobic (silica without O_2 treatment) sides over a 5-day period. Contact angle measurements were taken at the same spot for each side to assess wettability. Error bars represent s.d. of the mean from five different spots on a PET film.

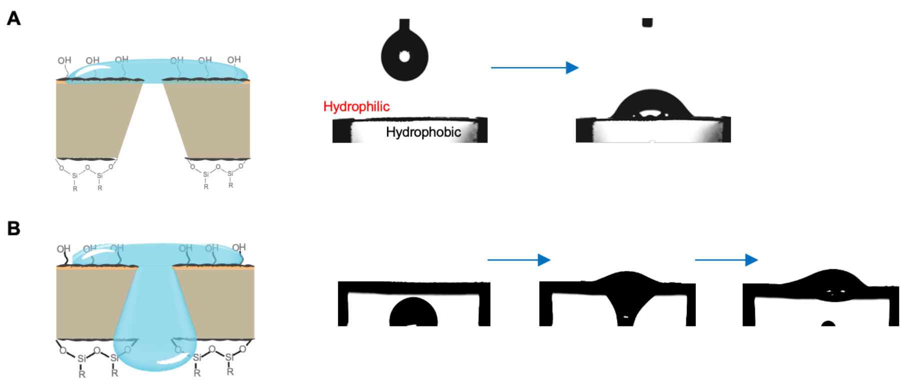

Fig. S13. Unidirectional transport on the Janus membrane. (**A** and **B**) Water transport evaluated on the Janus membrane under two conditions: a water droplet applied to the hydrophilic side with gravity (**A**), and a water droplet applied to the hydrophobic side (**B**).

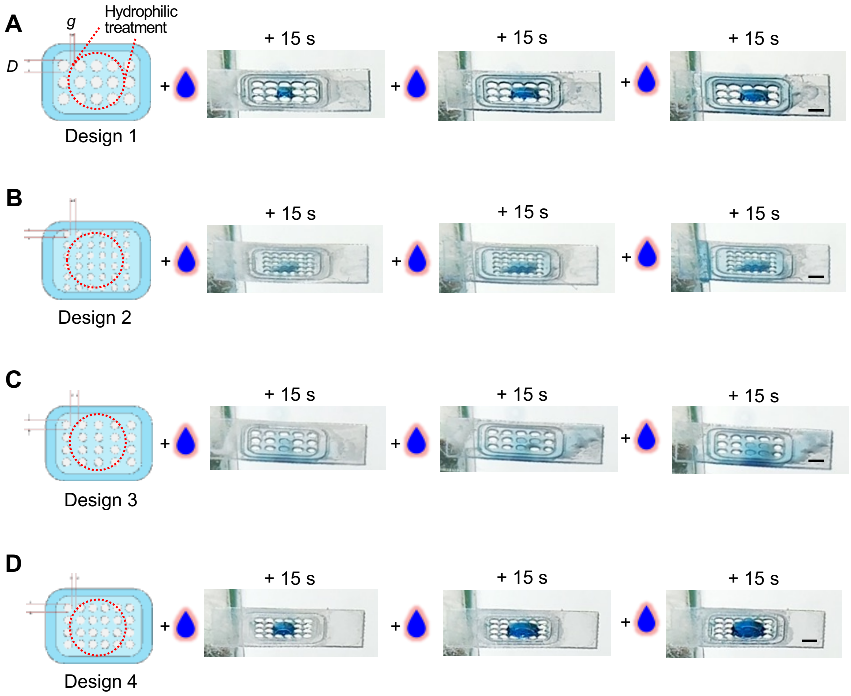

Fig. S14. Comparison of Janus membrane of unidirectional transport performance. (**A** to **D**) Unidirectional flow transport through Janus membranes demonstrated by applying 0.1 µl dyed ink three times in the hydrophobic side using a 0.48 mm diameter needle under four different designs (1 — 4); design 1: Hole diameter (*D*) 270 µm, gap (*g*) 200 µm, hole-to-gap ratio (*r*) 1.35 (**A**), design 2: $D = 140$ µm, $g = 250$ µm, $r = 0.56$ for design 2 (**B**), design 3: $D = 210$ µm, $g = 340$ µm, $r = 0.62$ (**C**), and design 4: $D = 210$ µm, $g = 160$ µm, $r = 1.31$ (**D**). Scale bars, 2 mm.

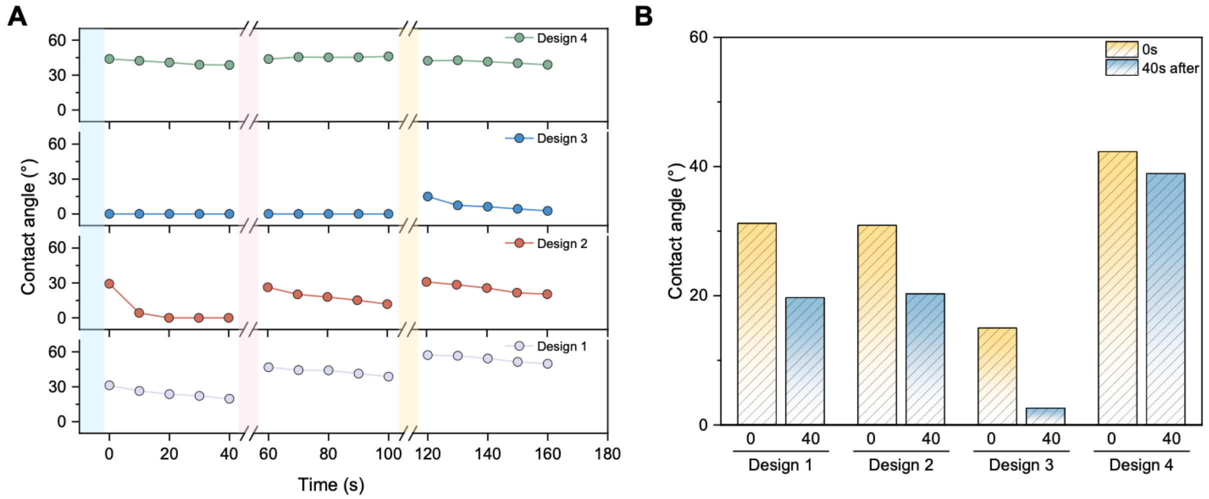

Fig. S15. Janus membrane performance evaluation. (**A**) Meniscus formation and contact angle change upon three times addition of water droplets from the anti-gravity direction, shaded regions indicate water droplet addition. (**B**) Contact angle change upon fixed-volume water droplet addition and 40 s after across 4 designs of Janus membrane parameters.

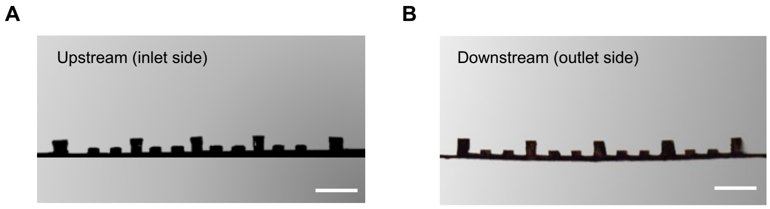

Fig. S16. Microscopic images of upstream and downstream of hierarchically graded microchannel for BMS^3. (**A** and **B**) Optical cross-sectional images of the upstream region of the inlet-side (**A**) and the outlet-side (**B**) microchannel. Scale bars, 200 µm.

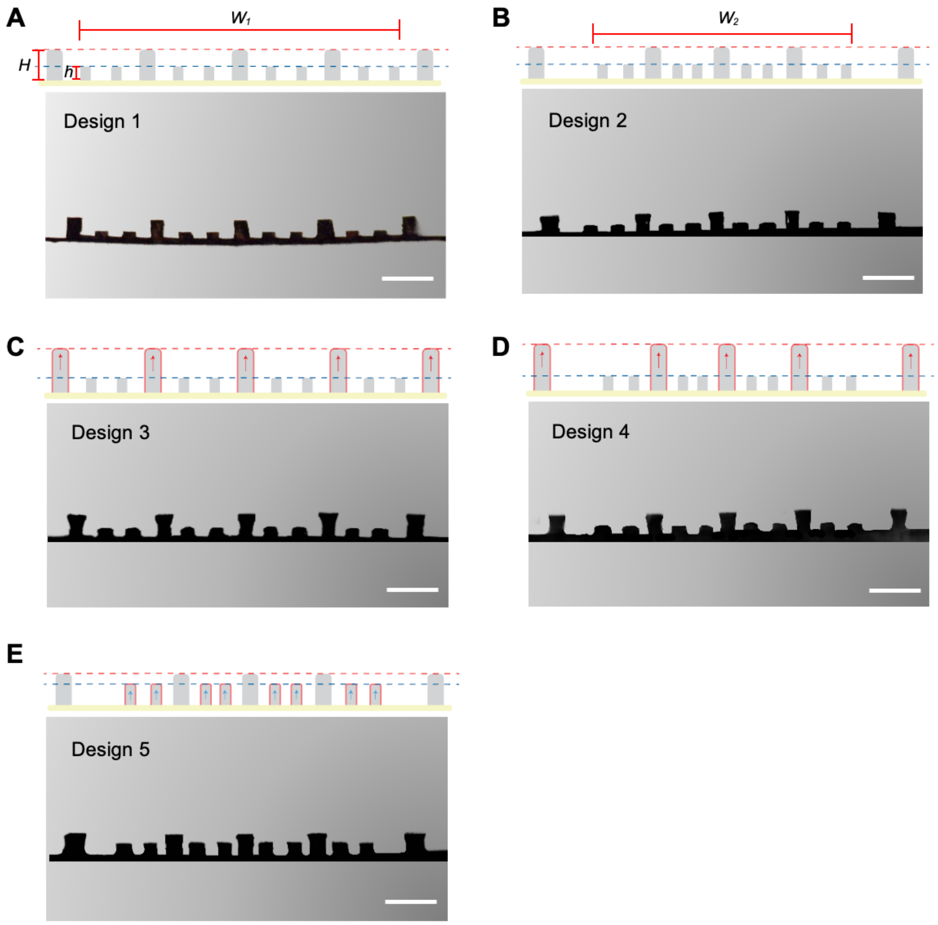

Fig. S17. Comparison images of microchannels. (**A** to **E**) Optical cross-sectional images of the upstream region of microchannels (Designs 1–5) with schematics to define major (H) and minor rib (h), without (W_1) and with width gradient ($W_2 = 0.9\ W_1$). Design 1: H = 14 µm and h = 5 µm with W_1 (**A**); Design 2: H = 14 µm and h = 5 µm with W_2 (**B**); Design 3: H = 17 µm and h = 5 µm with W_1 (**C**); Design 4: H = 17 µm and h = 5 µm with W_2 (**D**); and Design 5: H = 14 µm and h = 8 µm with W_2 (**E**). Scale bars, 200 µm.

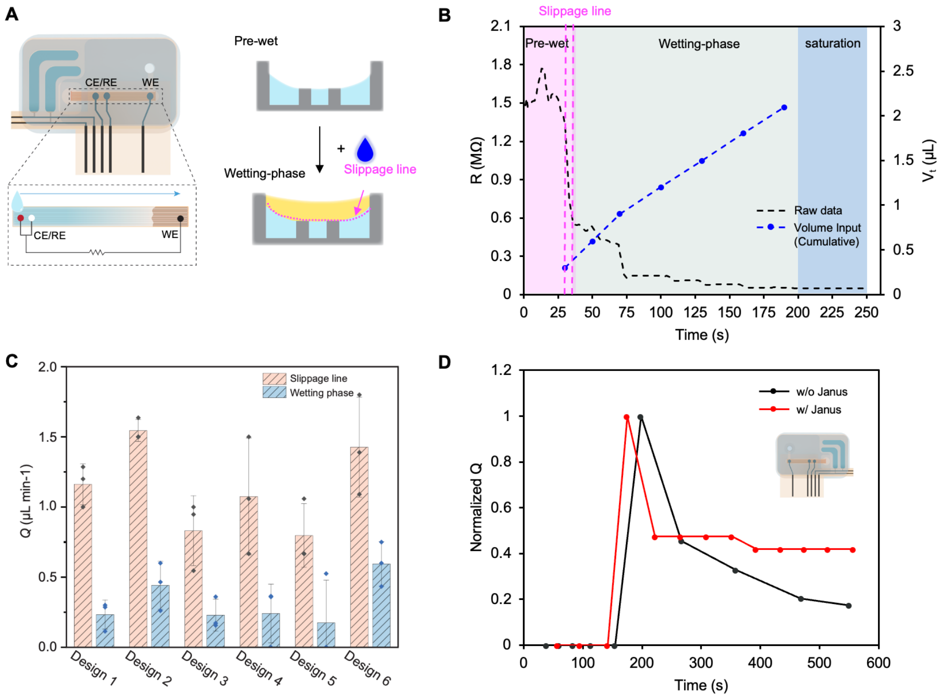

Fig. S18. Microchannel performance evaluation across five conditions. (**A**) Schematics of water transport phases in microchannel (pre-wet, slippage line, wetting phase, and saturation). (**B**) Impedance plot example (from one condition) illustrating phase definitions used across five conditions: slippage line (max $|\Delta R|$, $\Delta R < 0$), wetting (sustained $\Delta R < 0$), and saturation ($\Delta R \approx 0$). (**C**) Bar plot of flow rate (Q) at slippage line and during wetting phases for five microchannel designs (Designs 1–5) and Design 6 (Design 2 with Janus outlet). Error bars represent s.d. of the mean from 3 samples. (**D**) Normalized flow rate over time to assess Janus outlet performance. Each curve is scaled to its initial flow rate at the onset of wetting.

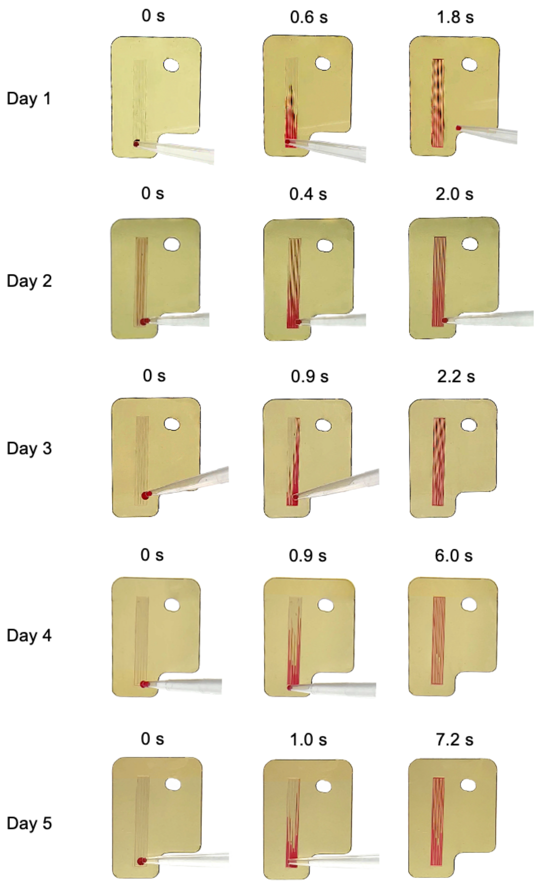

Fig. S19. Unidirectional transport and wettability of the hierarchically graded microchannel. Unidirectional flow and wettability were measured over a 5-day period using a single sample after O_2 treatment. Droplets (2×0.5 µl red dye and 2×0.5 µl DI water) were applied against gravity, and their interaction with the microchannel surface was recorded. Images show the first droplet test of each day.

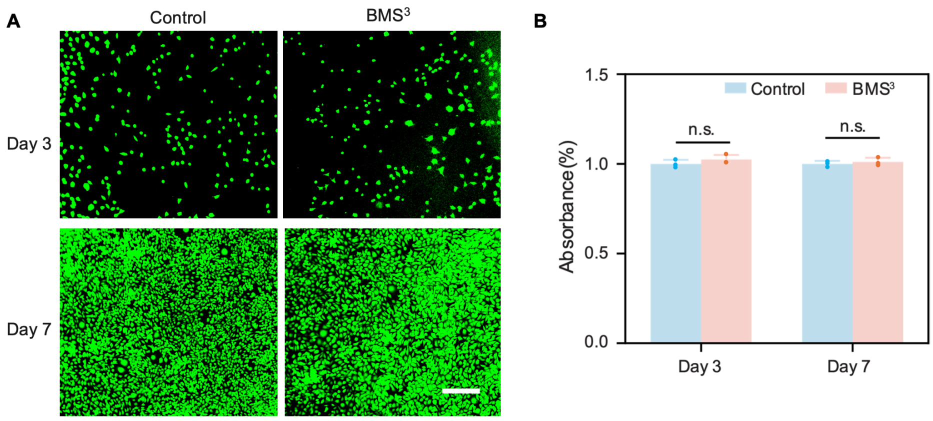

Fig. S20. **Biocompatibility of BMS^3 system evaluation in vitro.** **(A)** Fluorescence images of live/dead staining of L929 murine fibroblasts for 3 and 7 days of exposure to BMS^3 patch extract and control. Scale bar, 200 µm. **(B)** Absorbance measurement of cell viability of Day 3 and Day 7. Error bars represent s.d. of the mean from 3 measurements. Analysis by Student's t-test., n.s., not significant.

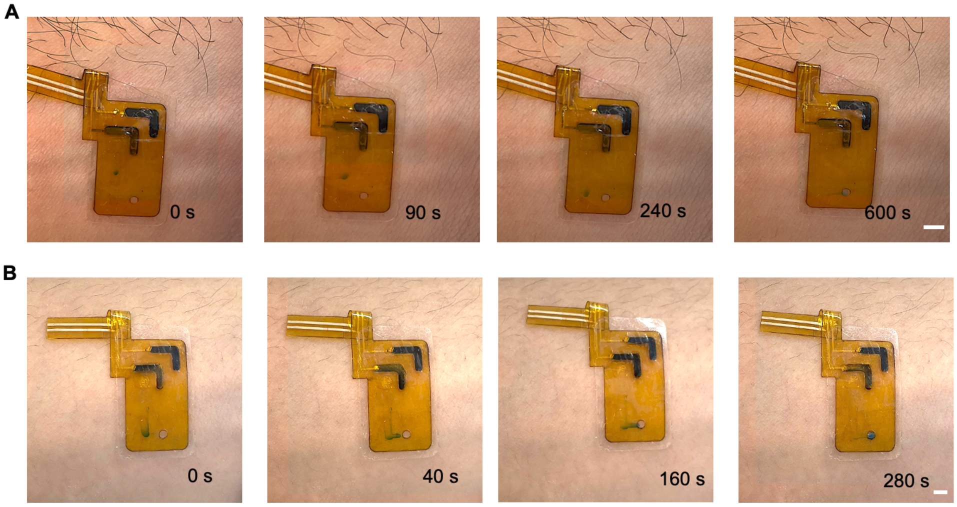

Fig. S21. Microfluidic sampling of sweat induced by the miniaturized IP module. (**A** and **B**) Microfluidic sweat sampling on two healthy subjects. A current of 100 µA was applied for 6 minutes using the wearable sensor patch to induce sweat. To visualize sweat flow, a blue dye was introduced into the microfluidic channel. Scale bars, 5 mm.

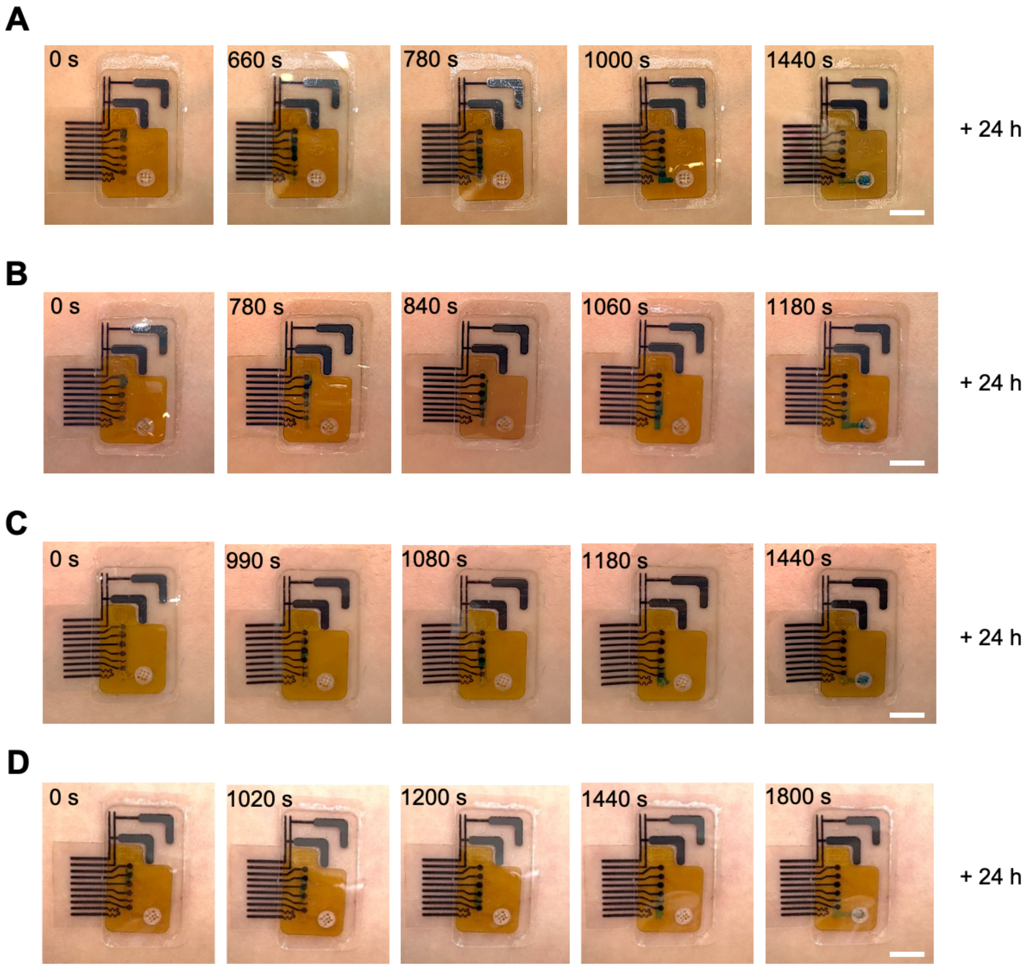

Fig. S22. Microfluidic sweat sampling 24-hour post iontophoresis. (**A** to **D**) Microfluidic sweat sampling on 4 healthy subjects. IP (100 µA for 6 minutes) was applied 24 hours prior to the flow test using the wearable sensor patch. A blue dye was introduced into the microfluidic channel to visualize sweat flow. Scale bars, 5 mm.

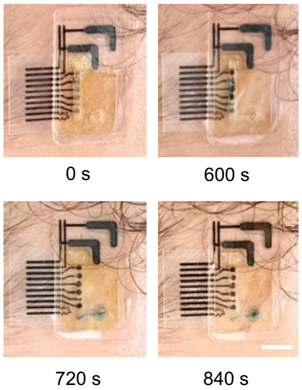

Fig. S23. Sweat flow during exercise session. Exercise began immediately after patch placement for the flow assessment. To visualize sweat movement, a blue dye was injected into the microfluidic channel. Scale bars, 5 mm.

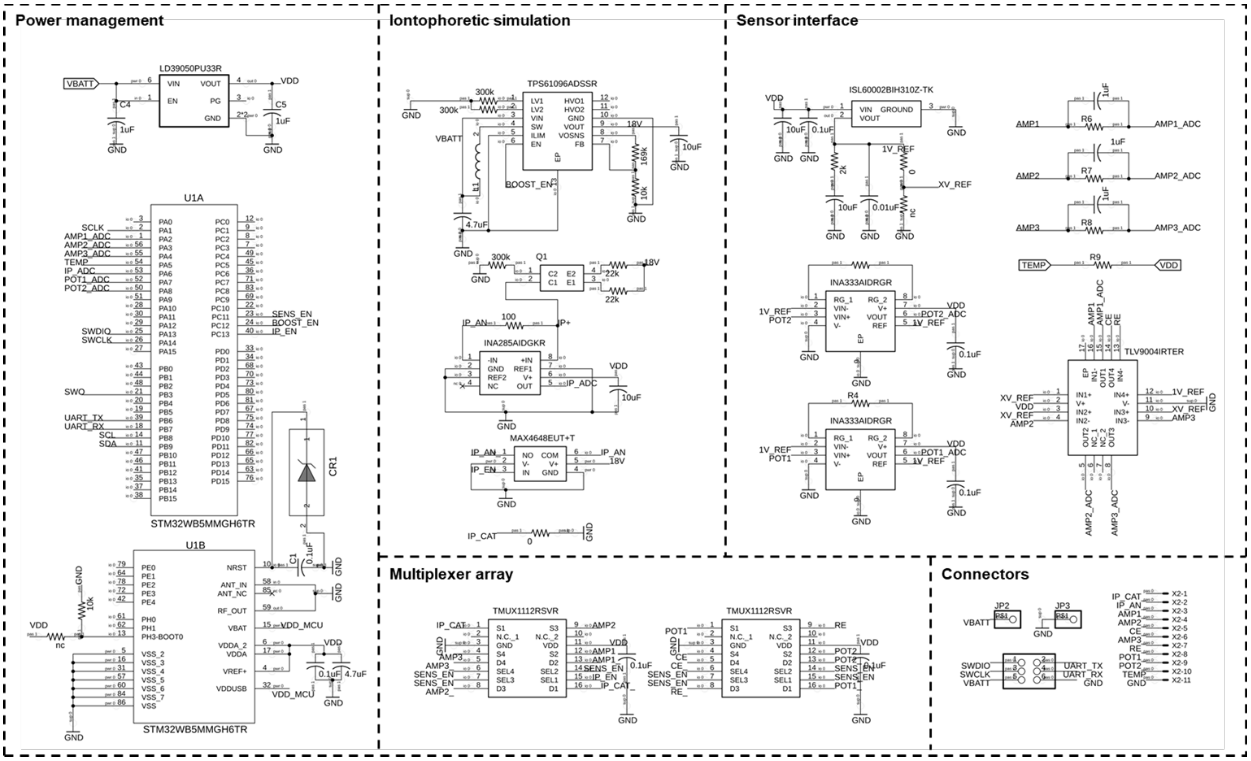

Fig. S24. Circuit diagram of the fully integrated BMS[3].

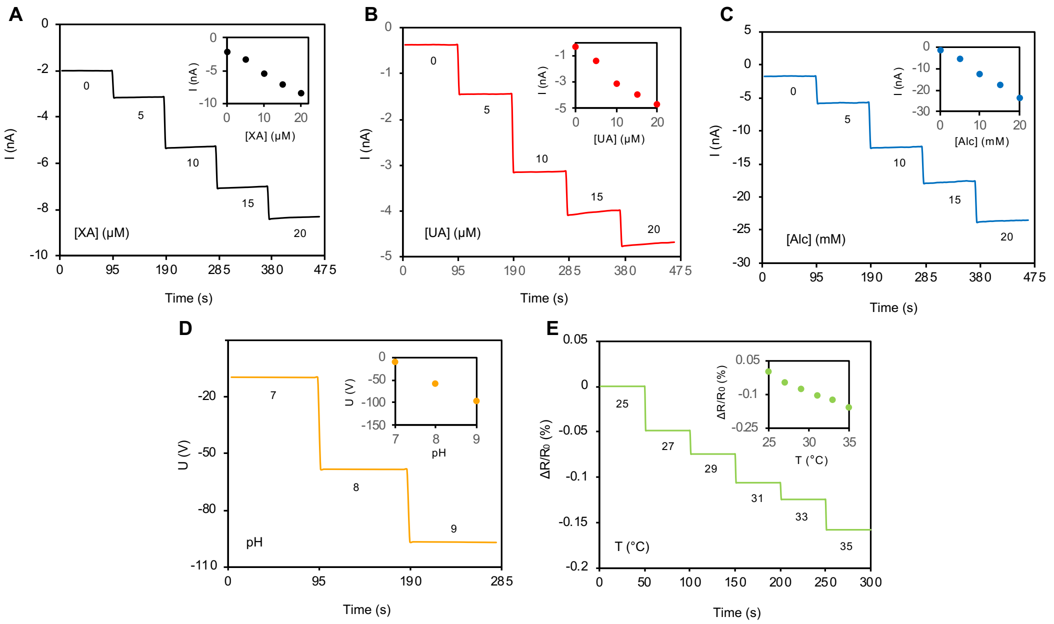

Fig. S25. Calibration of the microfluidic BMS^3 sensor arrays with PCB. (**A** to **E**) The PCB-recorded electrochemical response of xanthine (**A**), uric acid (**B**), alcohol (**C**), pH (**D**), and temperature (**E**) sensors in the microfluidics. Detection was performed under a controlled flow rate of 0.2 µl min^{-1}.

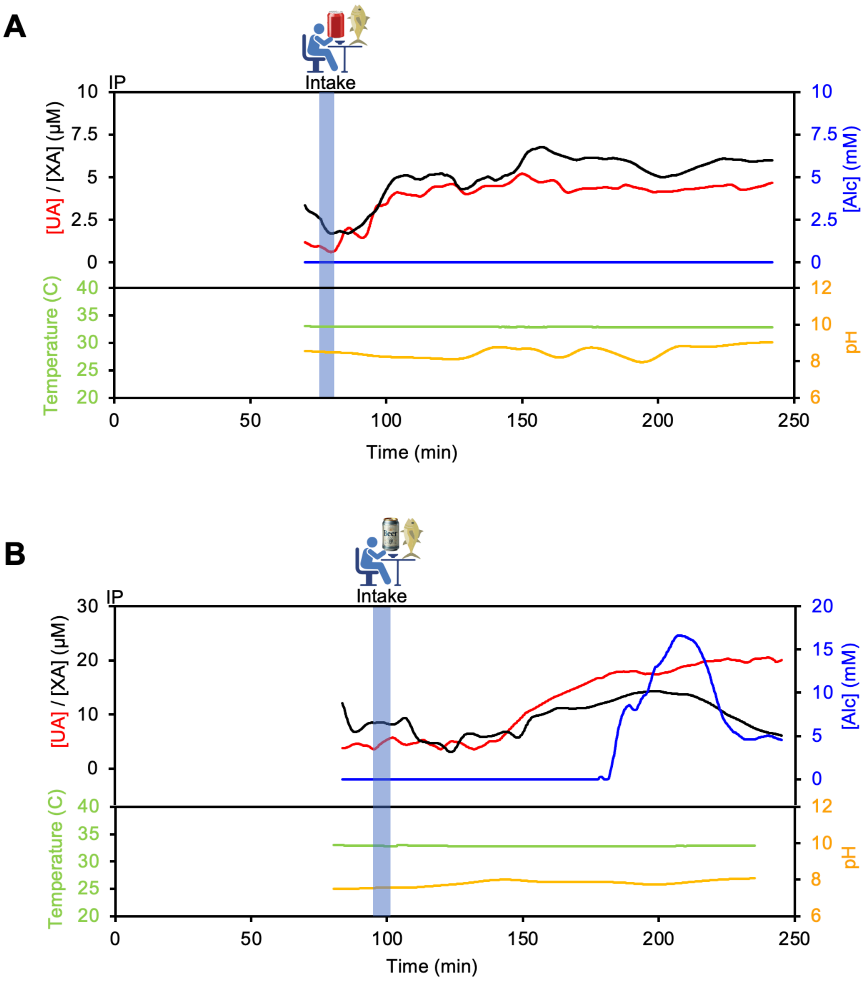

Fig. S26. On-body evaluation of BMS[3] on the healthy participants. On-body tests were conducted over 2.5 hours to assess the detection of purine metabolites—uric acid, xanthine, and alcohol—beyond the peak sweat induction phase (0–1 h post - IP). To evaluate real-time metabolic responses, dietary intake was introduced mid-session, with participants consuming either tuna with a soft drink (**A**) or tuna with beer (**B**), allowing for dynamic tracking of biomarker fluctuations.

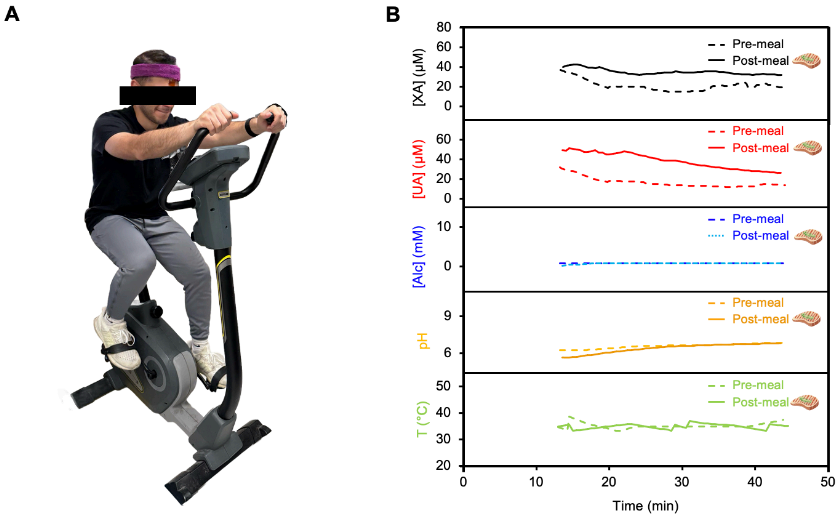

Fig. S27. On-body evaluation of exercise-induced sweat for dynamic biochemical monitoring. (**A**) Photograph of a participant wearing the BMS^3 patch during an on-body test. (**B**) Real-time monitored profiles of purine metabolites in a participant during exercise, comparing pre- and post-high purine food intake.

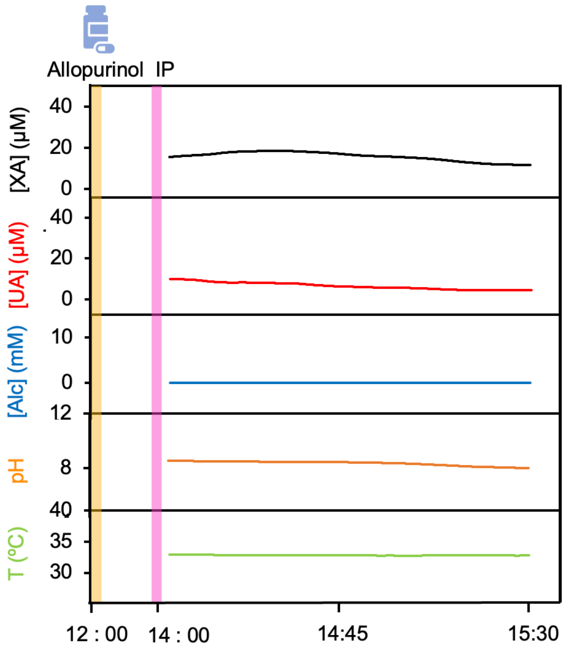

Fig. S28. On-body evaluation of BMS^3 on a patient with gout. The same participant as in Fig. 4J was instructed to take allopurinol 2 h before the on-body test.

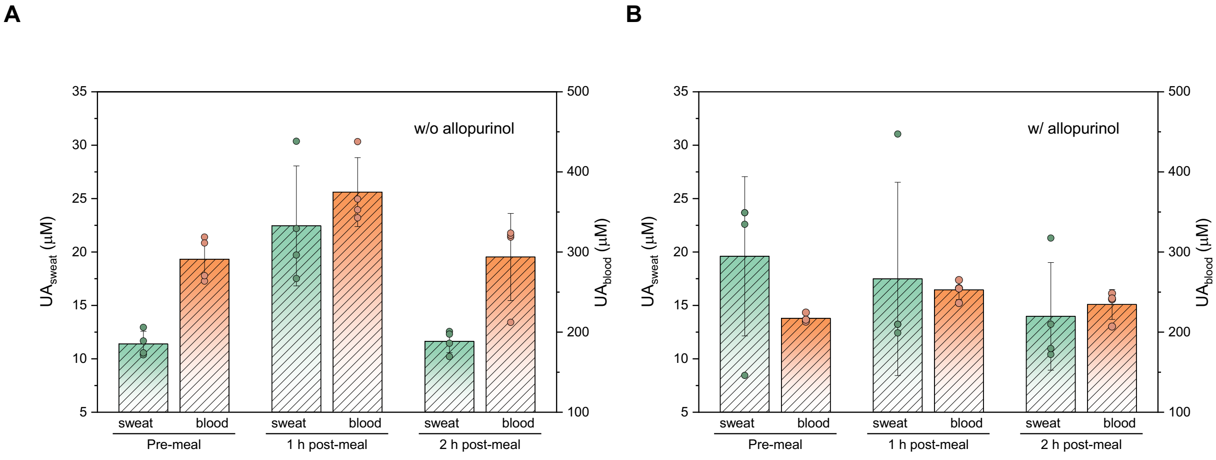

Fig. S29. On-body evaluation of sweat and blood correlation for purine metabolites under dietary intervention with BMS³. (**A** and **B**) Blood and sweat uric acid correlations in patients without allopurinol (**A)** and patients with allopurinol treatment (**B**). While wearing the BMS³ patch, participants consumed a high-purine meal (sardines and soda) and, if under treatment, also took allopurinol. Sweat uric acid levels were continuously monitored via the patch, while serum uric acid levels were measured via standard blood draws using a commercial assay kit. Error bars represent s.d. of the mean from 4 points at each session: pre-meal, 1 h post-meal (0.5–1 h after meal), and 2 h post-meal (1.5–2 h after meal).

Movie Captions

Movie S1. Water droplet interaction with superhydrophobic and superhydrophilic surfaces of Janus membranes.

Movie S2. Unidirectional antigravity water transport in hierarchically graded microchannels.

Movie S3. Microfluidic sweat sampling at 48 hours post-iontophoresis.
